# Investigating the pharmacodynamic durability of GalNAc–siRNA conjugates

**DOI:** 10.1093/nar/gkaa670

**Published:** 2020-08-18

**Authors:** Christopher R Brown, Swati Gupta, June Qin, Timothy Racie, Guo He, Scott Lentini, Ryan Malone, Mikyung Yu, Shigeo Matsuda, Svetlana Shulga-Morskaya, Anil V Nair, Christopher S Theile, Karyn Schmidt, Azar Shahraz, Varun Goel, Rubina G Parmar, Ivan Zlatev, Mark K Schlegel, Jayaprakash K Nair, Muthusamy Jayaraman, Muthiah Manoharan, Dennis Brown, Martin A Maier, Vasant Jadhav

**Affiliations:** Alnylam Pharmaceuticals, Inc., Cambridge, MA 02142, USA; Alnylam Pharmaceuticals, Inc., Cambridge, MA 02142, USA; Alnylam Pharmaceuticals, Inc., Cambridge, MA 02142, USA; Alnylam Pharmaceuticals, Inc., Cambridge, MA 02142, USA; Alnylam Pharmaceuticals, Inc., Cambridge, MA 02142, USA; Alnylam Pharmaceuticals, Inc., Cambridge, MA 02142, USA; Alnylam Pharmaceuticals, Inc., Cambridge, MA 02142, USA; Alnylam Pharmaceuticals, Inc., Cambridge, MA 02142, USA; Alnylam Pharmaceuticals, Inc., Cambridge, MA 02142, USA; Alnylam Pharmaceuticals, Inc., Cambridge, MA 02142, USA; MGH Program in Membrane Biology, Division of Nephrology, Massachusetts General Hospital and Harvard Medical School, Boston, MA 02114, USA; Alnylam Pharmaceuticals, Inc., Cambridge, MA 02142, USA; Alnylam Pharmaceuticals, Inc., Cambridge, MA 02142, USA; Alnylam Pharmaceuticals, Inc., Cambridge, MA 02142, USA; Alnylam Pharmaceuticals, Inc., Cambridge, MA 02142, USA; Alnylam Pharmaceuticals, Inc., Cambridge, MA 02142, USA; Alnylam Pharmaceuticals, Inc., Cambridge, MA 02142, USA; Alnylam Pharmaceuticals, Inc., Cambridge, MA 02142, USA; Alnylam Pharmaceuticals, Inc., Cambridge, MA 02142, USA; Alnylam Pharmaceuticals, Inc., Cambridge, MA 02142, USA; Alnylam Pharmaceuticals, Inc., Cambridge, MA 02142, USA; MGH Program in Membrane Biology, Division of Nephrology, Massachusetts General Hospital and Harvard Medical School, Boston, MA 02114, USA; Alnylam Pharmaceuticals, Inc., Cambridge, MA 02142, USA; Alnylam Pharmaceuticals, Inc., Cambridge, MA 02142, USA

## Abstract

One hallmark of trivalent *N*-acetylgalactosamine (GalNAc)-conjugated siRNAs is the remarkable durability of silencing that can persist for months in preclinical species and humans. Here, we investigated the underlying biology supporting this extended duration of pharmacological activity. We found that siRNA accumulation and stability in acidic intracellular compartments is critical for long-term activity. We show that functional siRNA can be liberated from these compartments and loaded into newly generated Argonaute 2 protein complexes weeks after dosing, enabling continuous RNAi activity over time. Identical siRNAs delivered in lipid nanoparticles or as GalNAc conjugates were dose-adjusted to achieve similar knockdown, but only GalNAc–siRNAs supported an extended duration of activity, illustrating the importance of receptor-mediated siRNA trafficking in the process. Taken together, we provide several lines of evidence that acidic intracellular compartments serve as a long-term depot for GalNAc–siRNA conjugates and are the major contributor to the extended duration of activity observed *in vivo*.

## INTRODUCTION

The recent regulatory approval of the first two RNAi therapeutics, ONPATTRO^®^ (patisiran) and GIVLAARI^®^ (givosiran), are the culmination of two decades of research and development following the initial discovery of RNAi by Fire and Mello (1–5). ONPATTRO^®^ is a lipid nanoparticle (LNP)-formulated small interfering RNA (siRNA) while GIVLAARI^®^ is a GalNAc-conjugated siRNA. Conjugation of oligonucleotides to GalNAc facilitates hepatocyte uptake through interaction with the asialoglycoprotein receptor (ASGPR) and is the preferred method for hepatocyte-specific delivery (6–8). Advances in siRNA design that chemically stabilize the duplex against nucleolytic cleavage have enabled subcutaneous (SC) injection of GalNAc–siRNA conjugates, removing the previous need for LNP formulation to shield the siRNA from degradation in the plasma (7,9,10). An early generation of GalNAc–siRNA conjugate (standard template chemistry, STC) was sufficiently stable to elicit *in vivo* activity, albeit requiring high doses (9,11). Further improvements in siRNA chemistry, including the addition of two phosphorothioate (PS) linkages on each of the 5′ ends of the siRNA guide and passenger strands, led to the enhanced stabilization chemistry (ESC) design. ESC siRNAs exhibit significantly enhanced efficacy and duration in preclinical species and in clinical trials (9). Finally, a comprehensive screening effort designed to optimize 2′-deoxy-2′-fluoro (2′-F) and 2′-OMe positions in both strands of the duplex led to the development of the Advanced ESC design (12). While these two designs are both extremely stable in *in vitro* metabolic stability assays, the Advanced ESC design is notable for its considerable potency and duration improvements over the ESC template *in vivo*.

To date, several investigational ESC and Advanced ESC GalNAc–siRNA conjugates evaluated in clinical trials have exhibited impressive pharmacodynamic properties, including sustained knockdown and months-long duration (13,14). The improvements in duration were also observed in preclinical species, including rodents and non-human primates (NHPs), suggesting that the biological mechanism underlying extended duration is conserved across species.

GalNAc–siRNA conjugates are rapidly taken up into clathrin-coated vesicles via high capacity ASGPR on the cell surface and traffic to endosomal compartments (15). As the pH of these compartments decreases, the GalNAc–siRNA is released from ASGPR, and the receptor can then recycle back to the cell surface. The siRNA conjugate remains in the maturing endosome as the pH continues to drop and the endosome transitions to a late endosome or multi-vesicular body. Despite rapid uptake and delivery to these subcellular organelles, previous reports suggest that <0.01% of GalNAc–siRNA is released into the cytosol from these compartments (15–17). Thus, endosomal escape is a significant rate-limiting step for oligonucleotide delivery efforts including antisense oligonucleotides (ASOs) and siRNAs. Endosomal escape can be enhanced using osmolytic agents such as chloroquine and nigericin, peptides that destabilize membranes, and endocytosis pathway disruptors (18–20). However, these techniques suffer from significant toxicity concerns and are not viable approaches for therapeutic applications.

Another delivery technique that enhances endosomal release in addition to shielding siRNA from nucleases is encapsulation into LNPs, which associate with ApoE in the blood and are recognized and internalized by low density lipoprotein (LDL) receptors and other ApoE-binding receptors on the surface of hepatocytes (21–23). LNPs and their cargo subsequently travel through the intracellular trafficking pathway and ultimately end up in late endosomes. Once in late endosomes, vesicle acidification leads to an interaction between the positively charged ionizable lipids on the LNP surface and anionic phospholipids in the endosomal membrane, facilitating membrane disruption and fusion with the LNP. These fusion events allow the siRNA cargo to be released into the cytosol where it is loaded into the RNA-induced silencing complex (RISC) (16,24). Despite the intrinsic endosome-escape mechanism of membrane fusion, siRNA escape from endosomal structures into the cytosol is still relatively low (1–2%), and limited to a small period of time in the course of endosome maturation (24). This percentage of release is equivalent to ∼2000–4000 siRNA molecules in the cytosol per cell, which is in line with estimated levels of active RISC following therapeutically relevant doses (24,25). Others have reported needing >5000 copies to achieve 80% silencing (16). Another important aspect of this delivery method is that over 70% of LNP-delivered siRNA is potentially exocytosed and lost due to association of lipids in the LNPs with protein factors that clear cholesterol from late endosomes to the plasma membrane (26).

Here, we consider three possible mechanistic explanations for the extended duration of GalNAc–siRNA activity observed *in vivo*: accumulation of siRNA at the site of subcutaneous injection, an extended half-life for RISC loaded with chemically stabilized siRNA, and an intracellular storage compartment that slowly releases functional siRNA for RISC loading over time. From our investigation we rule out the subcutaneous site of injection as a possible storage depot for siRNA as intravenously dosed GalNAc–siRNAs also show a similar durability profile. We compare siRNA delivery using different delivery methods (LNPs and GalNAc conjugates) to show that the half-life of siRNA-loaded RISC does not support the extended duration of activity. We determine that the metabolic stability of chemically modified siRNA is critical for potency and duration as it enhances survival in highly acidic subcellular compartments. Indeed, we demonstrate that functional siRNA can be liberated from acidic compartments up to three weeks post-dose and is loaded into newly synthesized Argonaute 2 (Ago2) several weeks after dosing. Our results support a model of slow GalNAc–siRNA release from highly acidic intracellular compartments into the cytosol where they are subsequently loaded into RISC (17,27). The ability of the siRNA to survive under these conditions, *i.e*. its metabolic stability, is directly correlated to the observed duration of effect. Therefore, improvements in siRNA design that enhance siRNA persistence against degradation in nuclease aggressive subcellular compartments lead to improved target knockdown and an extended duration of effect.

## MATERIALS AND METHODS

### Care and use of laboratory animals

All procedures using mice were conducted by certified laboratory personnel using protocols consistent with local, state, and federal regulations and in full compliance with AALAC guidelines at an AALAC-accredited facility. All procedures were approved by the Institutional Animal Care and Use Committee (IACUC) at Alnylam. All animals were acclimated in-house for 48 h prior to study start. Female C57BL/6 mice ∼6–8 weeks of age were obtained from Charles River Laboratories and randomly assigned to each group. All animals were treated in accordance with IACUC protocols. Mice were dosed subcutaneously at 10 μl/g with siRNA duplex, endolytic peptide, or phosphate buffered saline (PBS) control. siRNAs and the endolytic GalNAc peptide conjugates were diluted into PBS when making dosing solutions. All dosing solutions were stored at 4°C until 1 h before time of injection, when they were removed from storage and allowed to reach room temperature. Animals were sacrificed at days indicated in the figures, after which livers were harvested and snap frozen for further analysis. LNP formulations were dosed at a fixed volume of 100 μl by tail vein injection.

### Serum and plasma collection

Blood was collected utilizing the retro-orbital eye bleed procedure in accordance with IACUC approved protocols. For serum samples, blood was collected in Becton Dickinson serum separator tubes (Fisher Scientific, BD365967). Serum samples were kept at room temperature for 1 h and then spun in a micro-centrifuge at 21 000 ×*g* at room temperature for 10 min. Serum was transferred to 96-well plates for storage at −80°C. For plasma samples, blood was collected in Becton Dickinson plasma (K_2_EDTA) separator tubes (Fisher Scientific, BD365974). Plasma samples were kept at 4°C for no more than 30 min before being spun in a micro-centrifuge at 10 000 × *g* at 4°C for 10 min. Plasma was transferred to 96-well plates for storage at −80°C.

### Circulating protein level quantification

Factor 7 plasma protein levels were evaluated using an activity-based chromogenic assay (Biophen F7; Aniara). TTR serum protein levels were measured by ELISA (serum was diluted 1:4000 and used in a mouse prealbumin kit, ALPCO, 41-PALMS-E01). F12 plasma protein levels were measured by ELISA (plasma was diluted 1:20 000 and used in a mouse Factor 12 kit, Molecular Innovations, MFXIIKT-TOT).

### *In vivo* RNA extraction and RT-qPCR

Powdered liver (∼10 mg) was resuspended in 500 μl QIAzol (RNeasy 96 Universal Tissue Kit, Qiagen, 74881) and a 5 mm steel grinding ball was added to each sample. Samples were homogenized at 25/s for 1 min at 4°C using a TissueLyser II (Qiagen, 85300). Samples were incubated at room temperature for 5 min followed by addition of 100 μl chloroform. Samples were mixed by vigorously shaking the tubes, followed by a 10 min incubation at room temperature. Samples were spun at 12 000 × *g* for 15 min at 4°C and the supernatant was removed to a new tube and 1.5 volumes of 100% ethanol was added. Samples were then purified using a RNeasy 96 Universal Tissue Kit (Qiagen, 74881). Samples were eluted from RNeasy columns with 60 μl RNAse-free water (Ambion) and quantified on a Nanodrop (Thermo Fisher Scientific). 1.5 μg of RNA was used to generate cDNA using a High-Capacity cDNA Reverse Transcription Kit (Applied Biosystems, 4368813). qPCR reactions were performed using gene specific TaqMan assays for each target (Thermo Fisher, Mm00443267_m1 for *Ttr*, Mm00487329_m1 for *F7*, Mm01302526_m1 for *F9*) and mouse *Gapdh* as an endogenous control (Thermo Fisher, 4352339E). Real-Time PCR was performed in a Roche LightCycler 480 using LightCycler 480 Probes Master Mix (Roche, 04707494001). Data were analyzed using the ΔΔCt method normalizing to control animals dosed with PBS alone.

### Quantification of total liver siRNA levels and Ago2-loaded siRNA

Cohorts of mice were sacrificed on various days post-dose, and livers were snap frozen in liquid nitrogen and ground into powder for further analysis. Total liver siRNA levels were measured by Taqman PCR (SL-RT QPCR) as previously described (28–31). Ago2-bound siRNA from mouse liver was quantified as previously described (28–31). RT-qPCR primers and probes used in this study are summarized in Supplementary Table S1. For Ago-APP (affinity purification with peptides) the protocol has been previously described (32,33).

### *In vitro* gene expression and knockdown

#### Transfection

Primary mouse hepatocytes (PMH from BioIVT, M005052) were transfected with siRNA at multiple final concentrations (10, 2, 0.4, 0.08, 0.016, 0.0032, 0.00064 nM) to test for silencing efficiency at the time points of 2, 7 and 24 h. siRNA (5 μl) at the indicated concentrations was mixed with 4.9 μl of Opti-MEM (Gibco) and 0.1 μl of Lipofectamine RNAiMax (Invitrogen, Cat# 13778-150) per well of a 384-well plate and incubated at room temperature. After 15 min, 40 μl of BioIVT Invitrogro CP Rodent medium (Bioreclamation, Cat# Z99028) containing ∼5 × 10^3^ cells was added to the wells. Cells were incubated at 37°C for the times indicated above prior to RNA purification.

#### Free uptake

For time course evaluation of siRNA by ASGPR uptake in PMHs, siRNA at multiple final concentrations of 100, 20, 4, 0.8, 0.16, 0.032 nM (5 μl at the indicated concentration) was mixed with 5 μl of Opti-MEM per well of a 384-well plate. After 15 min, 40 μl of BioIVT Invitrogro CP Rodent medium containing ∼5 × 10^3^ cells was added to the wells. Cells were incubated at 37°C for 2, 7 and 24 h prior to RNA purification.

#### Total RNA isolation using Dynabeads mRNA isolation kit

Total RNA was isolated using an automated protocol on a BioTek 405 Select Microplate Washer using Dynabeads (Invitrogen, Cat # 10902D). Supernatant was discarded and 70 μl of lysis/binding buffer (100 mM Tris–HCl pH 7.5, 500mM LiCl, 10 mM EDTA pH 8.0, 1% LiDS, 5 mM DTT) was added. 10 μl of lysis buffer containing 3 μl of magnetic beads were added to each well. Plates were incubated on an electromagnetic shaker for 10 min at room temperature and then the magnetic beads were captured, and the supernatant removed. The bead-bound RNA was then washed once with 80 μl/well of Buffer A (10 mM Tris–HCl pH 7.5, 150 mM LiCl, 1 mM EDTA pH 8.0, 0.1% LiDS) and once with 80 μl/well of Buffer B (10 mM Tris–HCl pH 7.5, 150 mM LiCl, 1 mM EDTA pH 8.0) and twice with 80 μl /well of Buffer C (10 mM Tris–HCl pH 7.5).

#### cDNA synthesis using ABI high-capacity cDNA reverse transcription kit

cDNA synthesis was performed using an Applied Biosystems high capacity cDNA reverse transcriptase kit (4368813). To the wells of a 384-well plate containing the RNA isolated using Dynabeads was added 12 μl of a master mix containing 1.2 μl 10× Buffer, 0.4 μl 25× dNTPs, 1.2 μl 10× random primers, 0.6 μl reverse transcriptase, 0.6 μl RNase inhibitor and 8 μl of nuclease-free water. The plates were sealed, mixed, and incubated on an electromagnetic shaker for 10 min at room temperature, followed by 2 h at 37°C.

#### Gene expression analysis (RT-qPCR)

All probes for RNA quantification were acquired from Life Technologies utilizing their TaqMan gene expression system with dual-labeled probes. Target gene expression was normalized to *Gapdh* as a control in each well. Ct values were measured using a Light Cycler 480 (Roche). To calculate relative fold change real time data were analyzed using the ΔΔCt method and normalized to assays performed with cells treated with a non-targeting siRNA control. TaqMan probe catalogue numbers: Mouse *Gapdh* (4352339E), Mouse *Ttr* (Mm00443267_m1).

### FLAG-mAgo2 AAV

A 3xFLAG-tagged mouse Ago2 AAV (pAAV TBG-Flag3-mAgo2) was generated that expresses exogenous mouse Ago2 protein under control of the constitutively expressed liver-specific TBG promoter (Viral Vector Core, UMass Medical School). Animals were dosed with 1.0E+11 GC/mouse by tail vein injection in a total volume of 100 μl.

### Western blot for FLAG-mAgo2

Exogenous AAV-driven expression of FLAG-mAgo2 was verified in mice by Western blot analysis of liver lysates. Anti-FLAG M2 antibody (Sigma, F1804) was incubated with the Western membrane at 1:1000 overnight, followed by incubation with a goat anti-mouse secondary antibody (IRDye 800CW, Li-Cor, 925-32210) at 1:5000 for 1 h at room temperature.

### Fluorescence microscopy

Primary mouse hepatocytes were plated on an 8-well chamber slide coated with collagen (ibidi 8-well μ-slide, 70 000 cells/well). Alexa488-conjugated siRNA (siTTR-4) was incubated with primary mouse hepatocytes at 10 nM for free uptake delivery for 90 min or 16 h. Cells were treated with a lysosome staining agent (Abcam, Cytopainter Lysosomal Staining Kit – Red Fluorescence, 2 μl/ml for the 90 min time point and 1 μl/ml for the 16 h time point) for 20 min and live-cell images were taken after washing out the Cytopainter with cell culture media. Images were taken using a Nikon A1R confocal microscope equipped with a 60× objective (1.49 NA). The microscope was controlled by NIS-elements software.

### Ago2 half-life calculation

Drug effect was described as a direct inhibitory response model:

$$\begin{equation*}{\rm Drug}{\rm{\ }}{\rm effect}\ = \left( {1 - {I_{{\rm max}}}{\rm{\ }}\frac{{{C_{\left( t \right)}}}}{{{{\rm IC}_{50}} + {C_{\left( t \right)}}}}} \right)\ \end{equation*}$$

where *C*(*t*) is the predicted RISC-loaded concentration of siRNA at time *t*. I_max_ represents maximum inhibition effect of the siRNA targeting *Factor 7* and IC_50_ represents the RISC-loaded concentration of the drug in hepatocytes resulting in 50% of maximum inhibition of the targeting protein. Predicted RISC-loaded concentrations for different IC_50_ values are shown in Figure 5.

### LNP formulations

LNPs were prepared as previously described (34). Ionizable lipid DLin-MC3-DMA (MC3), 1,2-distearoyl-sn-glycero-3-phosphocholine (DSPC), cholesterol, and polyethyleneglycol dimyristoyl glycerol (PEG-DMG) were solubilized in ethanol and mixed at a molar ratio of 50:10:38.5:1.5 (MC3/DSPC/cholesterol/PEG-DMG) and used for a spontaneous vesicle formation process. The final N/P ratio was 3, corresponding to a total lipid to siRNA weight ratio of ∼9. siRNA was diluted to a final concentration of 1 mg/ml in 10 mM sodium citrate buffer (pH 4.0). LNPs were formed by microfluidic mixing of a final aqueous buffer containing RNA and ethanol containing lipids at a 3:1 ratio and 12 ml/min flow rate through syringe mixing (Precision Nanosystems, NanoAssemblr Benchtop Instrument). The ethanol was then removed via dialysis using Slide-A-Lyzers cassette (MWCO 20 kDa, ThermoFisher Scientific) in phosphate buffered saline (PBS, pH 7.2) overnight at room temperature. The resultant formulation was filtered using a 0.2 μm sterile polyethersulfone filter and stored at 2–8°C until use. The particle size of LNPs was measured using a Malvern Zetasizer Nano ZS (Malvern, UK) and the mean diameter was ∼65 nm (PDI 0.05) for all LNPs used in this study. Total siRNA concentration was determined by HPLC (A: 20 mM Tris buffer, 10 mM NaClO_4_·H_2_O, 1 mM EDTA and 50% MeCN, B: 20 mM Tris buffer, 800 mM NaClO_4_·H_2_O, 1 mM EDTA and 50% MeCN). Encapsulation efficiency of the LNPs was determined by Quant-IT Ribogreen (Invitrogen, Carlsbad, CA, USA) in the presence or absence of 2% Triton X-100 (Sigma-Aldrich). The encapsulation efficiency of all LNPs was around 97%.

### Endolytic peptide and GalNAc conjugate synthesis

#### GalNAc INF7 conjugate

Compound **800** synthesis was described previously (7). Compound **801** was purchased from Chem-Impex. Both D and L versions of peptide **803** were purchased from Bachem. Analytical HPLC was run on a Waters Alliance 2695 using Empower2 software, and a Waters Xbridge BEH C18, 130A, 3.5 uM 2.1 × 100 mm column. The reverse phase purification was run on an AKTA Explorer 100 FPLC using Unicorn 5.2 software, and a Waters Xterra Prep RP18 OBD, 125A, 5 uM 30× 100 mm column.

Compound **(802)**: Compound **800** (500 mg, 0.327 mmol) was dissolved in 100 ul of DMSO. In a separate vial, dissolved *N*-succinimidyl-3-(2-pyridyldithio)propionanate (SPDP) (122 mg, 0.392) in 100 ul of DMSO. Added solution of **800** to the solution of **801** and let the reaction stir at room temperature overnight. Cold ether was added to the reaction mixture to precipitate the product, which was carried out 2× times. The precipitate was centrifuged to a pellet, the ether decanted off and solid dissolved in water, frozen and lyophilized. This was used in next step without further purification. Mass calc. for C_75_H_128_N_12_O_29_S_2_: 1726.02, found: 1750.084 (M+Na) MALDI (MALDI date file in Supplemental Methods).

**Scheme 1.**
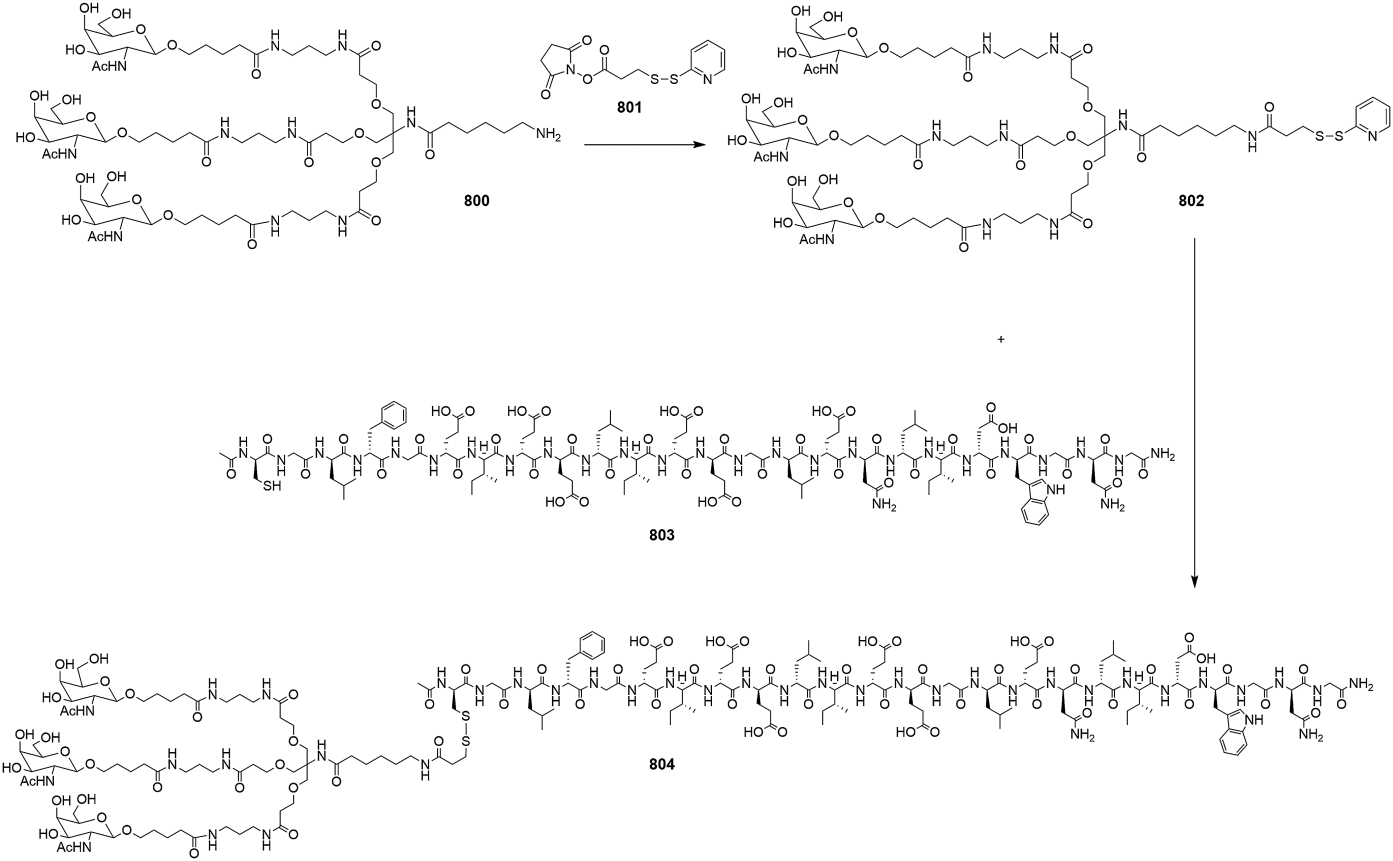
Generation of the GalNAc-conjugated D-INF7 peptide (Compound **804**).

Compound **(804)**: Compound **802** (80 mg, 0.03 mmol) was dissolved in 100 μl of DMSO. Then added solution of **802** to dry **803** (156 mg, 0.09 mmol) and let the reaction stir at room temperature overnight. The reaction was checked by HPLC which indicated the reaction was complete. The crude reaction mixture was purified by reverse phase purification using a C18 reverse phase column, eluting 5–80% acetonitrile (0.05% TFA)/ water (0.05% TFA). Mass calc. for C_189_H_303_N_39_O_70_S_2_: 4305.83, found: 4329.858 (M+Na) MALDI (MALDI data file in Supplemental Methods) (Scheme 1).

#### Oligonucleotide synthesis

Oligonucleotides were synthesized on a Bioautomation Mermade 12 Synthesizer using commercially available RNA amidites, 5′-*O*-(4,4′-dimethoxytrityl)-2′-deoxy-2′-fluoro- and 5′-*O*-(4,4′-dimethoxytrityl)-2′-*O*-methyl-3′-*O*-(2-cyanoethyl-*N*,*N*-diisopropyl) phosphoramidite monomers of uridine, 4-*N*-acetylcytidine, 6-*N*-benzoyladenosine and 2-*N*-isobutyrylguanosine using standard solid-phase oligonucleotide synthesis protocols. The GalNAc ligand was covalently linked to the 3′-end of the sense (S) strand of the siRNA by a phosphodiester linkage between the proline scaffold and the first nucleotide. Phosphorothioate linkages were introduced by sulfurization of phosphite linkages utilizing 0.1 M 3-((*N*,*N*-dimethyl-aminomethylidene)amino)-3*H*-1, 2, 4-dithiazole-5-thione (DDTT) in pyridine.

After synthesis, the support was dried under vacuum, and then added to a sealable container and heated at 60°C in aqueous ammonium hydroxide (28–30%) with 5% (v/v) of diethylamine for 5 h. After cooling to RT, the oligonucleotide was filtered to remove the support with 5× volume of water and analyzed by LC–MS and ion-exchange HPLC.

siTTR-4 was synthesized as mentioned above with a 5′ terminal trifluoroacetate protected amine. The oligo was cleaved from the solid support by shaking in AMA (50/50 (v/v) aqueous ammonium hydroxide (28–30%) and aqueous methylamine (40% wt.) for 3 h at RT. After standard purification as described above, 1.7 μmol of amine functionalized oligo was dissolved in 0.5 ml water. 200 μl of pH 7.2 0.2 M phosphate buffer was added followed by 5 mg Alexa488 NHS ester in 0.5 ml MeCN. The reaction was shaken for 12 h at RT wrapped in foil. It was then purified again by the techniques described above while taking care to minimize light exposure.

After deprotection and crude quality confirmation, ion-exchange HPLC purification was performed. Purification buffer A consisted of 20 mM sodium phosphate, 15% MeCN, pH 8.5 and Buffer B was the same composition with an additional 1 M sodium bromide. TSKgel Super Q-5PW (20) anion exchange resin from Tosoh Corporation (Cat# 0018546) was used for purification and a general purification gradient of 15–45% in ∼20 column volumes was applied. Fractions were analyzed by ion-exchange analysis using a Dionex DNAPac PA200 ion-exchange analytical column, 4mm x 250mm (ThermoFisher Cat# 063000) at room temperature. Buffer A was 20 mM sodium phosphate, 15% acetonitrile, pH 12 and Buffer B was identical with additional 1 M sodium bromide. A gradient of 30–50% over 12 min with a flow rate of 1 mL/min was used to analyze fractions. Fractions with greater than 85% purity were pooled, concentrated, and desalted over size exclusion columns (GE Healthcare Cat# 17-5087-01) with a flow rate of 10 ml/min.

## RESULTS

### GalNAc–siRNA trafficking delays the onset of RNAi activity *in vitro*

**Figure 1.**
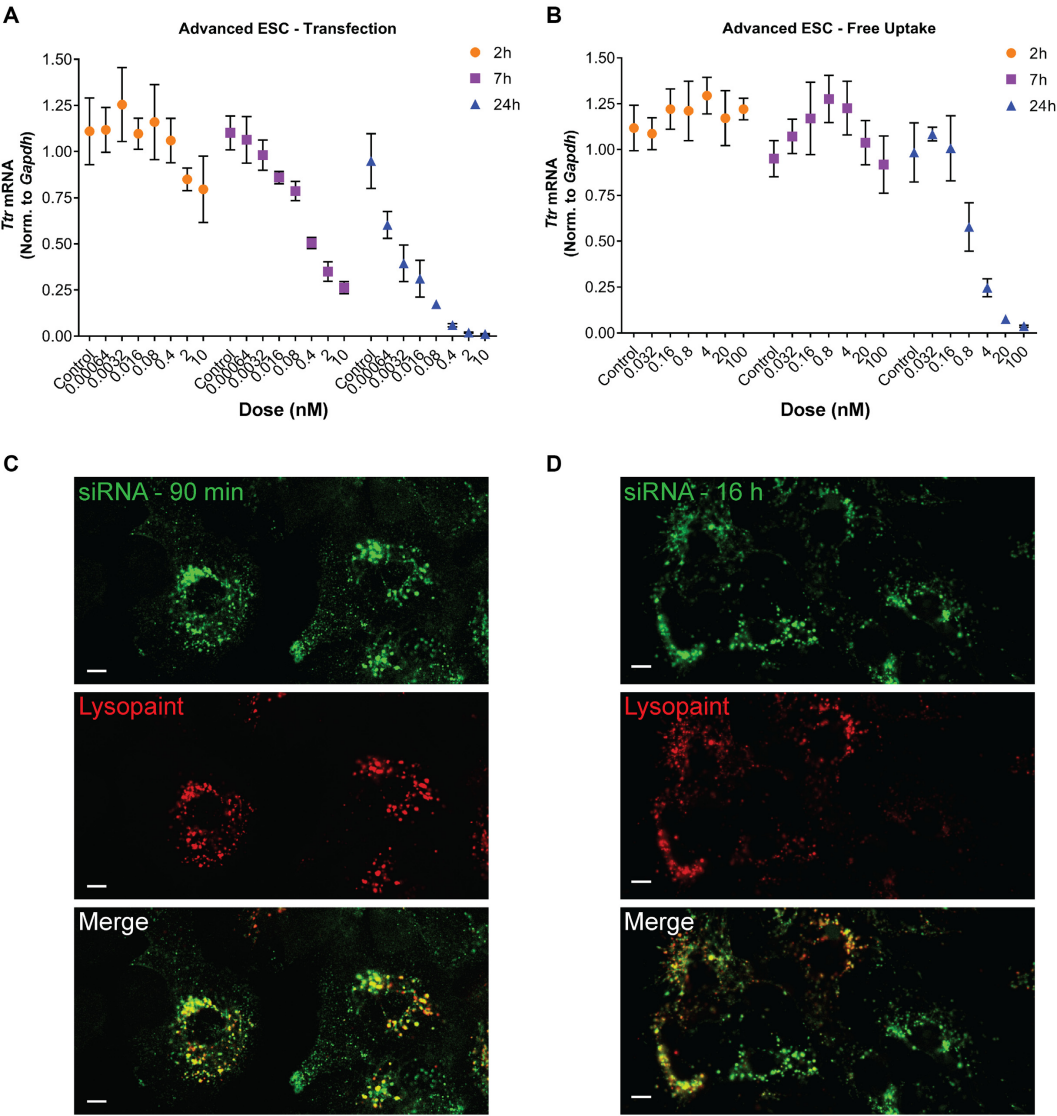
RNAi activity is delayed upon free-uptake of GalNAc–siRNAs. Advanced ESC GalNAc–siRNA-mediated knockdown (siTTR-2) of *Ttr* mRNA in primary mouse hepatocytes (PMH) following transfection (**A**) or free uptake (**B**) relative to *Gapdh*. A dose response and time course were carried out for all conditions. Similar results were obtained for an ESC GalNAc–siRNA design (siTTR-1, Supplementary Figure S1). Data is represented as mean ± SD, for *N* = 3. (C, D) An Advanced ESC GalNAc–siRNA labeled with Alexa-488 (siTTR-4, green, 5′ end of the sense strand), was incubated with primary mouse hepatocytes at 10 nM for free uptake delivery. Cells were treated with Lysosome Cytopaint (red) 90 min (**C**) or 16 hours (**D**) after siRNA treatment and live-cell images were taken 20 min later. Scale bars are 10 μm.

While previous work has shown that endosomal escape is a rate-limiting step for the activity of GalNAc–siRNA conjugates it remains unclear if endosomal escape significantly contributes to the long duration of activity observed for GalNAc–siRNAs (15). To evaluate different modes of siRNA delivery we introduced ESC and Advanced ESC GalNAc–siRNA conjugates (Table 1) to primary mouse hepatocytes (PMH) by transfection or *via* free uptake by ASGPR-mediated endocytosis. Freshly-isolated PMHs express ASGPR and are therefore capable of recapitulating the *in vivo* uptake mechanism for GalNAc–siRNAs (35). Knockdown of a target mRNA encoding rodent *Transthyretin* (*Ttr*) was monitored at several timepoints following GalNAc–siRNA treatment. Both ESC and Advanced ESC GalNAc–siRNAs delivered by transfection led to rapid (<7 h) and robust knockdown of *Ttr* in PMHs (Figures 1A and Supplementary Figure S1A). In contrast, delivery of GalNAc–siRNA *via* free uptake significantly delayed the onset of knockdown (between seven and 24 h, Figures 1B and Supplementary Figure S1B), despite rapid (<90 min) internalization to acidic compartments in primary mouse hepatocytes, as shown by colocalization with Lysosome Cytopaint, a marker of acidic endolysosomal compartments (Figure 1C). The significant delay between internalization of GalNAc–siRNAs and the downstream RNAi activity upon free-uptake but not transfection-mediated delivery is consistent with previous data suggesting that subcellular trafficking is a rate-limiting step for GalNAc–siRNA-mediated knockdown (15).

**Table 1.** Designs and sequences of GalNAc–siRNA conjugates

Compound	Design	Strand	Sequence (5′-3′)	Calculated MW	Observed MW
siTTR-1	ESC	S	A•a•CaGuGuUCUuGcUcUaUaAL	8590.169	8589.17
		AS	u•U•aUaGaGcAagaAcAcUgUu•u•u	7595.937	7594.58
siTTR-2	Advanced ESC	S	a•a•caguGuUCUugcucuauaaL	8686.456	8684.42
		AS	u•U•auaGaGCaagaAcAcuguu•u•u	7632.043	7631.5
siTTR-3	Advanced ESC	S	a•a•caguGuUCUugcucuauaaL	8686.456	8684.42
		AS	u•U•auaGagcaagaAcAcuguu•u•u	7656.116	7655.51
siTTR-4	Advanced ESC	S	488a•a•caguGuUCUugcucuauaaL	9493.167	9493.35
		AS	u•U•auaGaGCaagaAcAcuguu•u•u	7632.043	7631.5
siF7-1	ESC	S	C•a•GgAuCaUCUcAaGuCuUaAL	8612.224	8611.32
		AS	u•U•aAgAcUuGagaUgAuCcUg•g•c	7626.95	7626.08
siF7-2	Advanced ESC	S	c•a•ggaucauCUcaagucuuaaL	8732.582	8731.73
		AS	u•U•aagAcUuGagaUgAuCcug•g•c	7651.021	7650.38
siF7-3	Advanced ESC	S	c•a•ggauCaUCUcaagucuuaaL	8708.511	8707.19
		AS	u•U•aagAcuUgagaUgAuccug•g•c	7675.093	7673.96
siF9-1	ESC	S	C•a•GuAcCuUAGaGuUcCaCuAL	8588.198	8586.51
		AS	u•A•gUgGaAcUcuaAgGuAcUg•a•a	7674.014	7672.19
siF9-2	Advanced ESC	S	c•a•guacCuUAGaguuccacuaL	8684.485	8683.71
		AS	u•A•gugGaACucuaAgGuacug•a•a	7710.122	7708.79
siF12-1	Advanced ESC	S	g•a•aacuCaAUAaagugcuuuaL	8756.561	8755.73
		AS	u•A•aagCacuuuauUgAguuuc•u•g	7610.037	7609.42

S and AS represent sense and antisense strands. Upper- and lower-case letters indicate 2′-deoxy-2′-fluoro (2′-F) and 2′-*O*-methyl (2′-OMe) ribosugar modifications, respectively. • indicates a PS linkage. L indicates a GalNAc ligand. 488 indicates an Alexa488 fluorophore.

### GalNAc–siRNA metabolic stability enhances target knockdown and extends duration of activity

**Figure 2.**
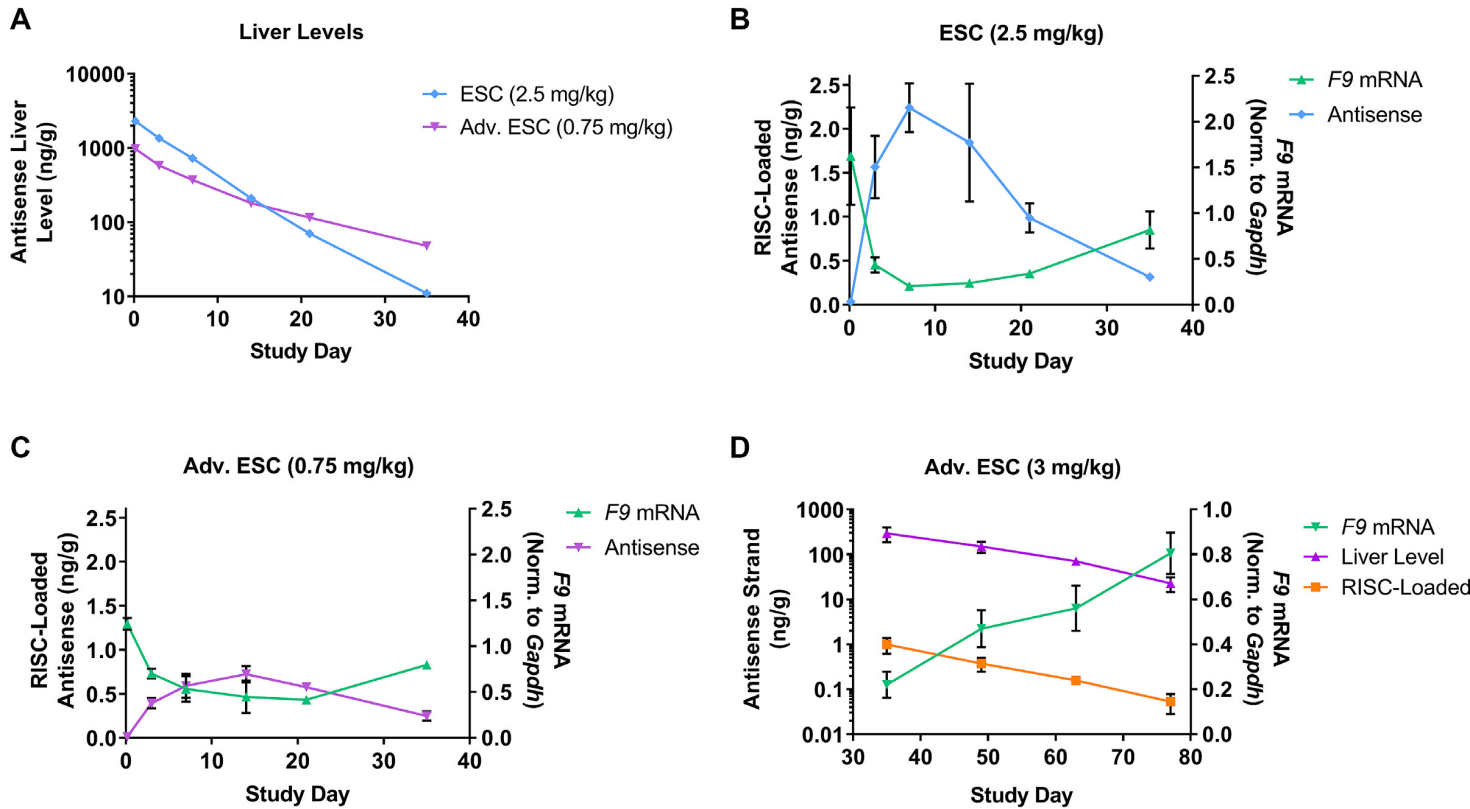
Increased siRNA stability improves knockdown and duration of activity. Cohorts of mice were dosed SC with ESC (2.5 mg/kg, siF9-1) or Advanced ESC (0.75 mg/kg, siF9-2) siRNA targeting *F9* (A–C). Animals were sacrificed after 4 hours and on days 3, 7, 14, 21 and 35 post dose. Antisense siRNA levels (μg/g, μg antisense strand per gram of liver) were measured from total liver (**A**) and plotted on a log10 scale. RISC-loaded antisense siRNA levels were also measured (ng/g, ng of antisense strand per gram of liver) and plotted for ESC (**B**) and Advanced ESC (**C**) siRNAs. *F9* mRNA knockdown was quantified and normalized to *Gapdh* for all samples (B, C). Data is represented as mean ± SD, for N = 3. Similar results were obtained with siRNAs targeting *Factor 7* (*F7*, Supplementary Figure S2). (**D**) Cohorts of mice were dosed with Advanced ESC (siF9-2, 3 mg/kg) siRNA targeting *F9*. Animals were sacrificed on days 35, 49, 63 and 77 post dose. Antisense siRNA liver levels were plotted on a log10 scale alongside RISC-loaded antisense siRNA levels (both shown in ng/g units, ng of antisense strand per gram of liver). *F9* mRNA knockdown was normalized to *Gapdh* for all samples. Data is represented as mean ± SD, for *N* = 3.

Next, we investigated the *in vivo* relationship between siRNA chemical stability and knockdown by examining siRNAs targeting *Factor 9* (*F9*) mRNA in the mouse liver. *In vivo* dose levels were adjusted to achieve a similar level of knockdown between the ESC and Advanced ESC designs. The ESC siRNA had higher initial liver levels due to a 3.3-fold higher dose but was more rapidly eliminated from the liver than the Advanced ESC siRNA, reflecting the lower stability of the ESC design (Figure 2A). Consistent with our previous findings for siRNAs targeting *Ttr*, the superior stability of the Advanced ESC siRNA increased the efficacy and duration of RNAi activity when the differences in dose are taken into account (Figure 2B, C) (12). Additionally, the enhanced stability of the Advanced ESC design extended the length of time the siRNA continually loaded into RISC, as the antisense strand of the ESC siRNA was maximally loaded into RISC at day 7, while the Advanced ESC siRNA was maximally loaded on day 14 (Figure 2B, C). The correlation between target knockdown and RISC loading was also observed for siRNAs targeting *Ttr* and *Tmprss6*, and both ESC and Advanced ESC designs targeting *Factor 9* and *Factor 7* (Figures 2 and Supplementary Figure S2) (12,36).

The relationship between RISC-loaded antisense strand and target knockdown was examined at later time points using a higher dose (3 mg/kg) of the *F9*-targeting Advanced ESC siRNA (Figure 2D). At this higher dose, there was still significant knockdown and RISC loading at later time points. Cohorts of animals were sacrificed starting at day 35 post-dose through day 77. The total antisense siRNA strand liver level at day 35 was in the same range as it was with the 0.75 mg/kg dose on days 3–21 previously (Figure 2D). This amount of siRNA supported significant RISC loading on day 35 (∼1 ng/g), which correlated with ∼70% *F9* knockdown. As antisense strand liver levels decreased through day 77, a concurrent decrease of antisense strand-loaded RISC was observed, which correlated with a recovery of *F9* mRNA levels back to baseline (Figure 2D). These results support the assertion that siRNA chemical stability significantly contributes to the efficacy and duration of activity. These results also argue against the possibility that an increase in duration is caused by an increase in RISC half-life, as the decrease in RISC-loaded antisense strand is highly correlated with the decrease in total antisense strand levels in the liver for ESC and Advanced ESC designs.

### The mode of delivery significantly impacts activity and duration of GalNAc–siRNAs

GalNAc–siRNA conjugates delivered via ASGPR-mediated endocytosis traffic through the endocytic pathway and accumulate in acidic intracellular compartments. The slow release from these compartments appears to delay the onset of activity and high metabolic stability is paramount for achieving maximal activity. We reasoned that siRNA stability may not play as critical a role when delivered by LNP, as the siRNA would be protected from the harsh environment of endolysosomal/acidic compartments. However, the duration of effect following LNP-mediated delivery should be reduced due to the one-time, bolus-like release of siRNA into the cytosol, which is markedly different than the slow release over time that is observed following conjugate delivery (16,24,37).

**Figure 3.**
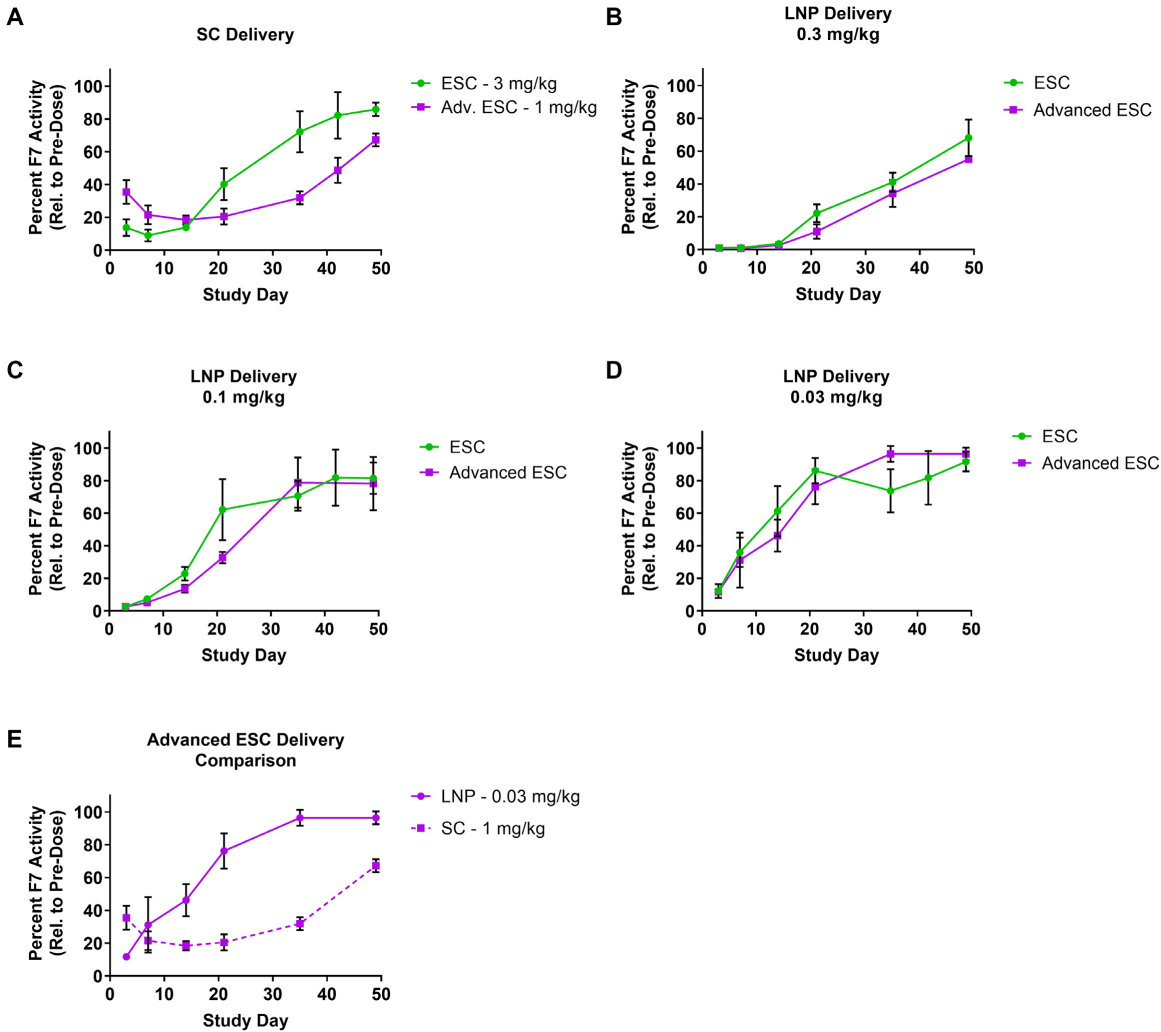
siRNA route of delivery affects target knockdown profile and duration of effect. (**A**) GalNAc–siRNAs were dosed SC at 3 mg/kg (ESC, siF7-1) or 1 mg/kg (Advanced ESC, siF7-3) and plasma F7 protein activity relative to pre-dose was measured. (**B–D**) ESC and Advanced ESC GalNAc–siRNAs were formulated into LNPs and delivered by IV at 0.3, 0.1 and 0.03 mg/kg. Both conjugate (SC) and LNP (IV) arms of the experiment were monitored for 49 days by measuring F7 protein activity in the blood. (**E**) A comparison between the Advanced ESC siRNA delivered as an LNP (0.03 mg/kg, siF7-3) or conjugate (1 mg/kg, siF7-3) is shown. Data is represented as mean ± SD, for *N* = 3.

To investigate this in more detail, we directly compared GalNAc-conjugate and LNP delivery for ESC and Advanced ESC siRNAs targeting *Factor 7* (*F7*) in mice. GalNAc–siRNAs were dosed subcutaneously (SC) at either 3 mg/kg (ESC) or 1 mg/kg (Advanced ESC) to achieve equivalent knockdown (Figure 3A, (12)). In parallel, ESC and Advanced ESC GalNAc–siRNAs were formulated into LNPs (21) and dosed intravenously (IV) at 0.3, 0.1 and 0.03 mg/kg (Figure 3B–D). F7 protein activity in the blood was monitored for 49 days in both conjugate (SC) and LNP (IV) arms of the experiment (Figure 3). SC-dosed ESC and Advanced ESC conjugates showed significantly different pharmacodynamic profiles (Figure 3A). Despite a 3-fold higher dose for the ESC conjugate resulting in a somewhat stronger effect at the time of maximum knockdown, the Advanced ESC conjugate demonstrated a longer duration of activity (Figure 3A). However, LNP delivery abolished the differences in potency and duration between ESC and Advanced ESC siRNAs (Figure 3B–D). Across all LNP dose groups, encompassing both conjugate designs, similar knockdown and duration of activity was observed with a slight improvement for the Advanced ESC, possibly due to increased cytosolic stability prior to RISC loading (Figure 3B–D). These data suggest that the inherent potency of the ESC and Advanced ESC siRNAs is largely similar, and that the observed discrepancy in their activity profiles, when delivered SC as GalNAc conjugates, is predominantly due to their metabolic stability in acidic subcellular compartments. Comparing the two delivery methods at similar levels of maximum knockdown, we observed a markedly different onset of activity. With SC-administered GalNAc–siRNA, maximal reduction of F7 protein activity was achieved between 7–14 days followed by an extended duration of effect compared to a quick onset of knockdown and reduced duration in animals dosed with LNPs (Figure 3E). The more rapid onset and decreased duration of RNAi activity by LNP-delivered siRNAs is consistent with its proposed mechanism of intracellular trafficking and enhanced endosomal escape mediated by the membrane disruption properties of LNPs at low pH. These results further support the hypothesis that endosomal release is a significant rate-limiting step for GalNAc-conjugated siRNA delivery.

**Figure 4.**
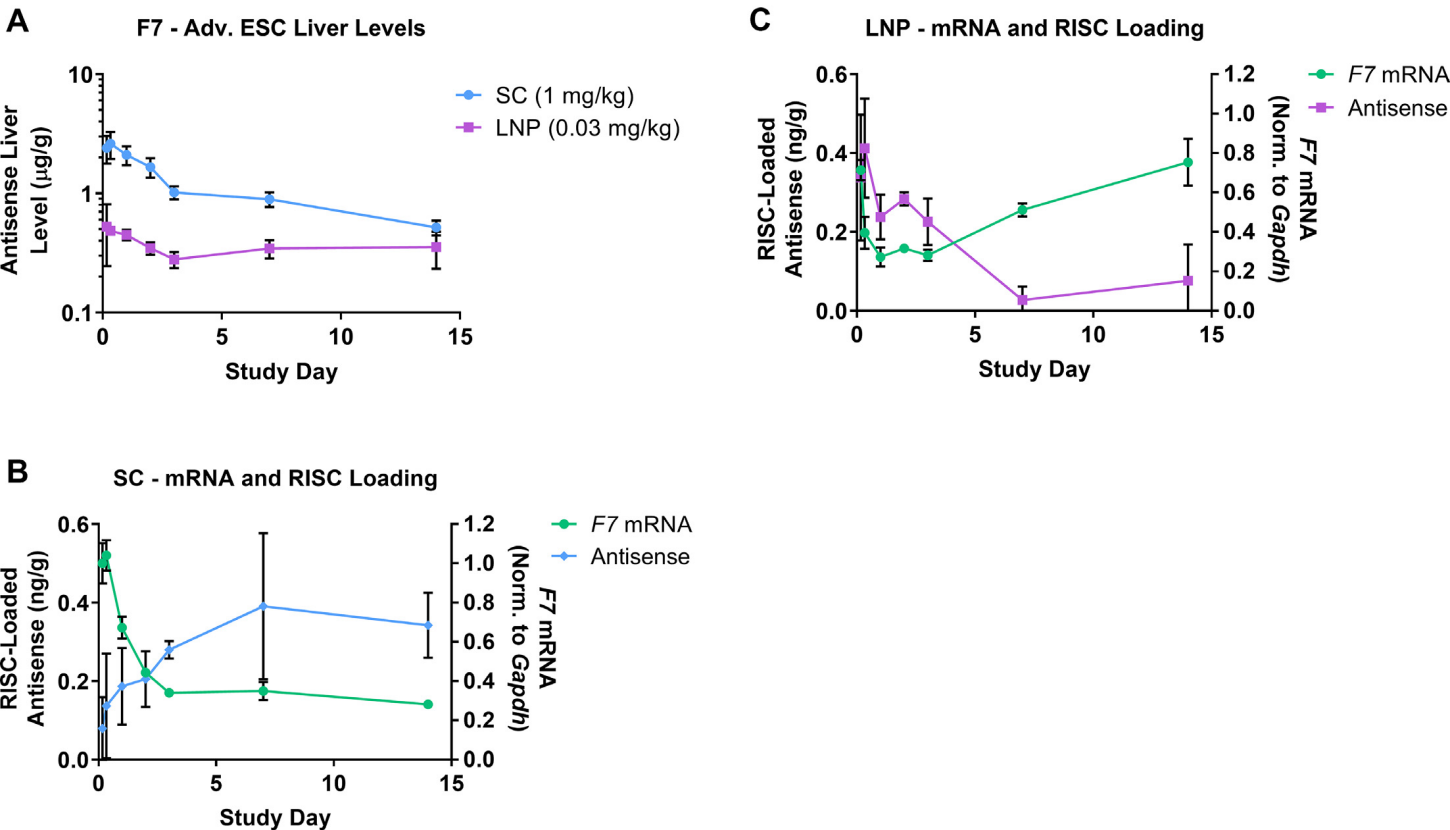
SC siRNA delivery supports sustained RISC loading compared with LNP delivery. (**A**) An Advanced ESC GalNAc–siRNA (siF7-3) targeting *F7* mRNA was delivered by IV (LNP, 0.03 mg/kg) or by SC injection (1 mg/kg) into cohorts of mice (*n* = 3). Animals were sacrificed at 4 and 8 hours, and days 1, 2, 3, 7 and 14 and antisense siRNA levels (μg/g, μg antisense strand per gram of liver) were measured from total liver. (B, C) RISC-loaded antisense siRNA levels were measured for SC (**B**) and LNP (**C**) groups (ng/g, ng of antisense strand per gram of liver). *F7* mRNA knockdown was quantified and normalized to *Gapdh* for all samples. Data is represented as mean ± SD, for *N* = 3.

We repeated the experiment shown in Figure 3E and measured *F7* mRNA knockdown, total liver siRNA levels and RISC loading at several early time points through day 14 (Figure 4). As expected from the different dose levels, total liver exposure levels were higher in the SC than the LNP group at early time points (Figure 4A). However, RISC-loaded antisense levels at the time of maximum *F7* knockdown were similar for both groups (∼0.3–0.4 ng/g, Figure 4B, C). Importantly, RISC loading persisted in the SC group but rapidly decreased in the LNP group after day 2 (Figure 4B, C). This observation supports the model that SC delivery establishes an intracellular depot of siRNA that is slowly released into the cytosol where it can engage with the RISC complex, while LNP-delivered siRNA is released as a bolus into the cytosol soon after delivery and does not establish a depot at the low dose levels tested herein. The apparent stabilization of total liver siRNA levels in the LNP group most likely represents siRNA that is trapped in subcellular compartments that are not release-competent (16,24).

**Figure 5.**
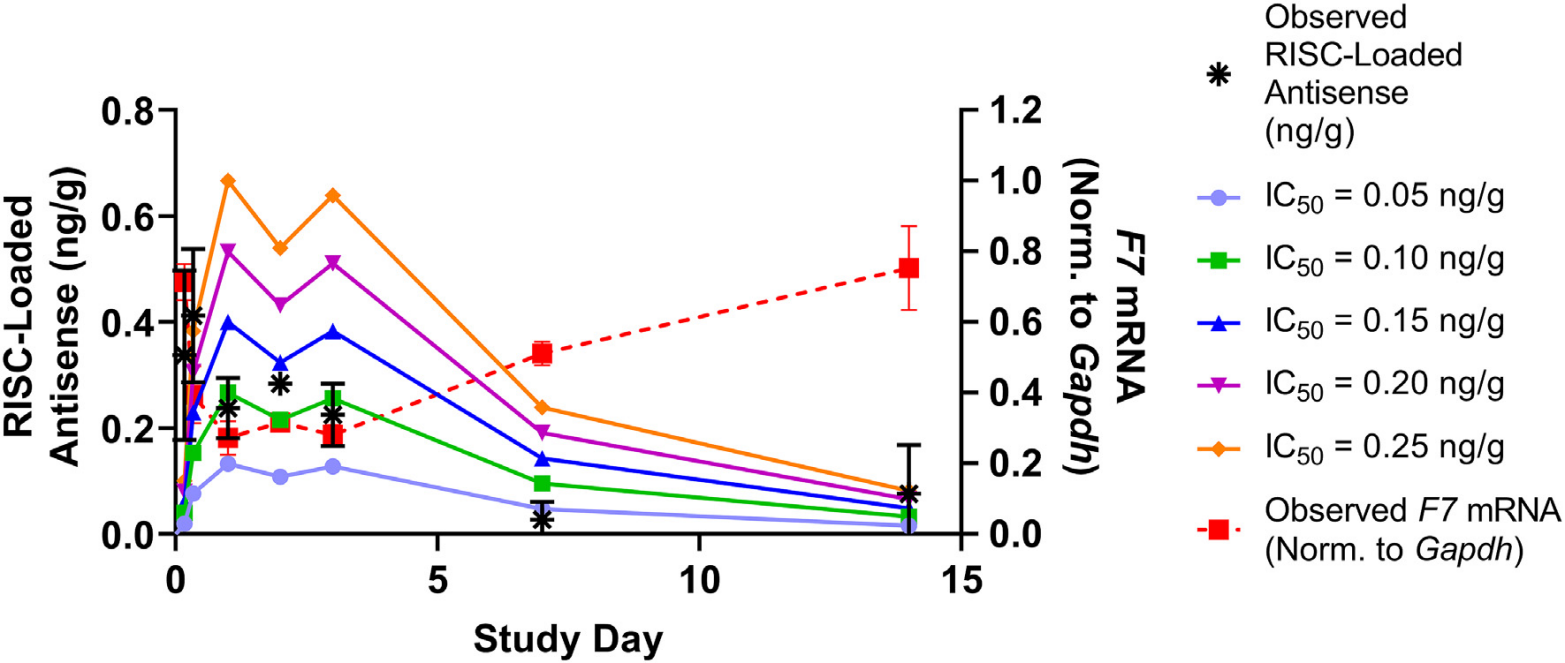
Estimate of antisense-loaded RISC half-life *in vivo*. Data from Figure 4C (advanced ESC, siF7-3, 0.03 mg/kg by LNP) were used to calculate the half-life of antisense-loaded RISC. The terminal half-life (*t*_1/2_) is independent of IC_50_ and is approximately 3.8 days. The IC_50_ is estimated to be between 0.05 and 0.15 ng/g (ng loaded antisense strand in RISC per gram of liver).

Together, these LNP experiments show that the delivery modality can have a significant impact on knockdown and duration of effect. ESC and Advanced ESC siRNAs that have comparable intrinsic potency behave similarly when delivered by LNP, suggesting that RISC loading and subsequent steps in the RNAi pathway are not significantly affected by differences in metabolic stability of the siRNA. These data also show that RISC-loaded siRNA has a finite half-life that cannot support prolonged target knockdown without being replenished (Figure 4B, C). We calculated the half-life of siRNA-loaded RISC using the 0.03 mg/kg LNP data from Figure 4C and derived a value of ∼3.8 days (Figure 5). This value is comparable to previous reports that determined a miRNA-loaded RISC half-life of 5–10 days (38). Thus, the half-life of siRNA-loaded RISC is likely insufficient to support the weeks- to months-long duration of effect observed in rodents, NHPs and humans following SC injection (12,14). However, the half-life of siRNA-loaded RISC in humans has not been experimentally determined outside of *in vitro* systems, so the possibility still exists that it may play a more substantial role in the extended duration in humans.

### The SC site of injection does not act as depot for the extended duration of effect

**Figure 6.**
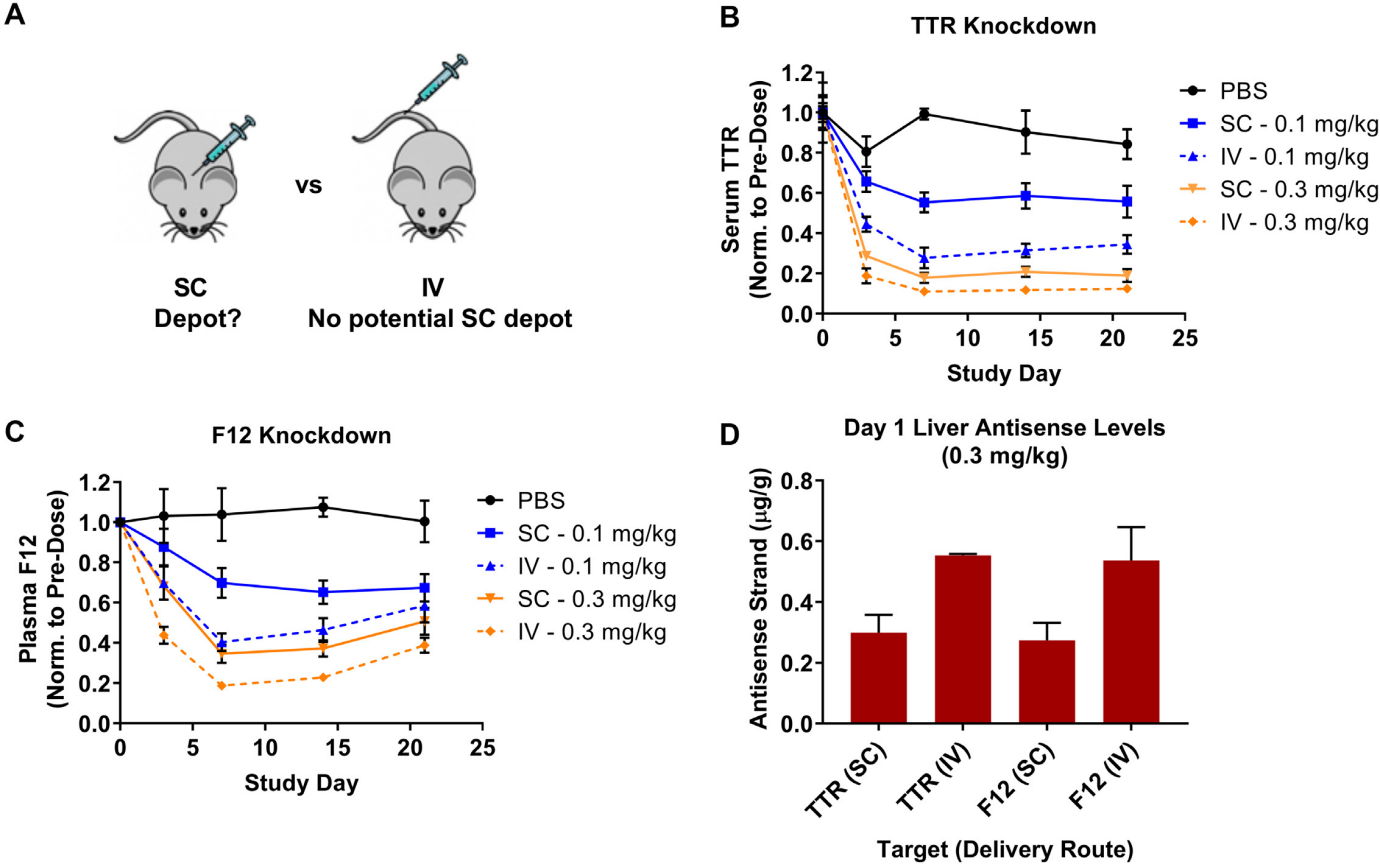
The SC site of injection is not a depot for GalNAc–siRNA conjugates. (**A**) Schematic illustrating the location of SC (back) and IV (tail) injection sites for the GalNAc–siRNA conjugates used in this comparison (note: IV-injected GalNAc–siRNA was not LNP-formulated). (B, C) Cohorts of mice (*n* = 3) were dosed by SC or IV injection at 0.3 or 0.1 mg/kg with Advanced ESC GalNAc–siRNA conjugates targeting *Ttr* (**B**, siTTR-2) or *F12* (**C**, siF12-1) mRNA. (**D**) Antisense siRNA levels (μg/g, μg antisense strand per gram of liver) were measured from total liver collected one day post-dose from the 0.3 mg/kg groups. The route of administration (RoA) is indicated. Data is represented as mean ± SD, for *N* = 3.

Our data suggest that siRNA conjugates delivered by SC injection establish an intracellular depot from which siRNA is slowly released over time, enabling continual loading into RISC. However, it may also be conceivable that a fraction of SC-dosed GalNAc–siRNA is sequestered in the SC space following delivery and is slowly released into the blood where it undergoes ASGPR-mediated uptake into hepatocytes at later times (Figure 6A). We tested this possibility by dosing Advanced ESC GalNAc–siRNA conjugates targeting rodent *Ttr* and *Factor 12* (*F12*) by SC or IV injection. IV-injected conjugates (not LNP formulated) do not have the opportunity to accumulate in the SC space as they are directly injected into the blood. Interestingly, IV-dosed Advanced ESC conjugates showed greater knockdown than those dosed by SC for both targets (Figure 6B, C). Importantly, total liver antisense siRNA levels were greater following IV dosing for both targets (Figure 6D). The superior knockdown seen following IV dosing does not support the hypothesis that the SC injection site could serve as a storage depot for GalNAc–siRNA conjugates.

### Chemically stabilized siRNAs persist in highly acidic subcellular compartments

GalNAc–siRNAs accumulate in acidic, endolysosomal compartments within hours of delivery in primary hepatocytes and *in vivo* (Figure 1C; (17,39)). Based on the lines of evidence already discussed, we hypothesize that Advanced ESC siRNAs survive the acidic, metabolically highly active conditions longer than the less stable ESC siRNAs and that this could be the main driver of potency and duration differences observed between the two designs. Because it is technically challenging to directly measure endolysosomal contents, we took advantage of a tri-GalNAc conjugated endolytic peptide (stabilized with D-amino acids) derived from the HA2 domain of the influenza virus (D-INF7, Supplemental Methods, (40,41)). Upon exposure to low pH (<5) the peptide adopts an alternate configuration and promotes membrane disruption, liberating the contents of acidic organelles, including siRNAs, into the cytosol (42–44). Enhanced target knockdown following peptide administration reflects the release of siRNA from acidic organelles and subsequent loading into RISC.

**Figure 7.**
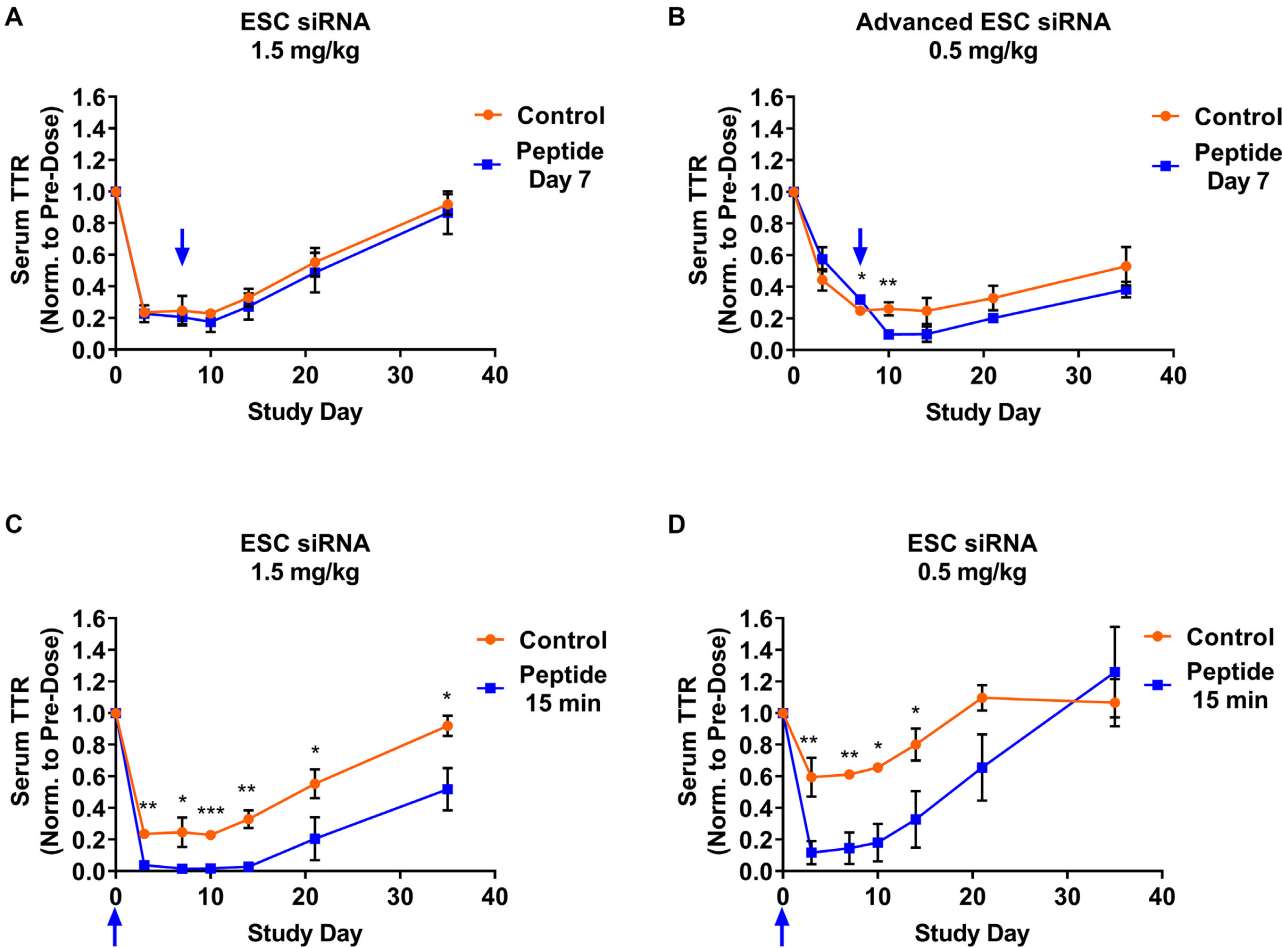
Functional siRNA can be liberated from acidic intracellular compartments by an endolytic GalNAc-peptide conjugate. Cohorts of control mice were dosed with an siRNA alone, either ESC (siTTR-1) or Advanced ESC (siTTR-3), targeting *Ttr*. Separate cohorts of mice were dosed with the same siRNA as control cohorts followed by the GalNAc-INF7 endolytic peptide at various time points. (**A**) siTTR-1 was dosed SC at 1.5 mg/kg in two cohorts of mice, one of those cohorts was also dosed with 5 mg/kg of the endolytic peptide at day 7 post siRNA dose. (**B**) siTTR-3 was dosed SC at 0.5 mg/kg in two cohorts of mice, one of those cohorts was also dosed with 5 mg/kg of the endolytic peptide at day 7 post siRNA dose. (**C**) siTTR-1 was dosed SC at 1.5 mg/kg in two cohorts of mice, one of those cohorts was also dosed with 5 mg/kg of the endolytic peptide 15 min post siRNA dose. Note the control siRNA group is the same in (A) and (C). (**D**) siTTR-1 was dosed SC at 0.5 mg/kg in two cohorts of mice, one of those cohorts was also dosed with 5 mg/kg of the endolytic peptide 15 min post siRNA dose. Serum TTR protein levels were monitored for 35 days post-dose. Blue arrows indicate endolytic peptide dose time. Data is represented as mean ± SD, for *N* = 3. Welch's independent t-test was utilized to compare the mean value between the control and peptide-treated groups at each timepoint to identify significant differences in target knockdown (*P ≤ 0.05, **P ≤ 0.01, ***P ≤ 0.001). Full results for the statistical analysis are included in the Supplemental Information.

We dosed animals with either ESC (1.5 mg/kg, Figure 7A) or Advanced ESC (0.5 mg/kg, Figure 7B) siRNA conjugates targeting *Ttr*, and subsequently dosed animals with 5 mg/kg of the GalNAc-INF7 peptide conjugate 7 days after the GalNAc–siRNA. The siRNA doses were selected to achieve similar maximum activity (∼80% knockdown) with both designs. Administration of the GalNAc–INF7 peptide conjugate 7 days after the siRNA significantly enhanced the knockdown of serum TTR by the Advanced ESC, but not the less stable ESC, conjugate (Figure 7A, B). These results suggest that the Advanced ESC siRNA survived the highly acidic environment for 7 days and was fully functional upon release into the cytosol. We also found that the GalNAc-INF7 peptide enhanced the potency of the less stable ESC siRNA if dosed 15 min after either a 1.5 or 0.5 mg/kg siRNA dose (Figure 7C, D), indicating that the ESC siRNA can survive the acidic environment for a shorter period of time and is fully functional if released early enough.

**Figure 8.**
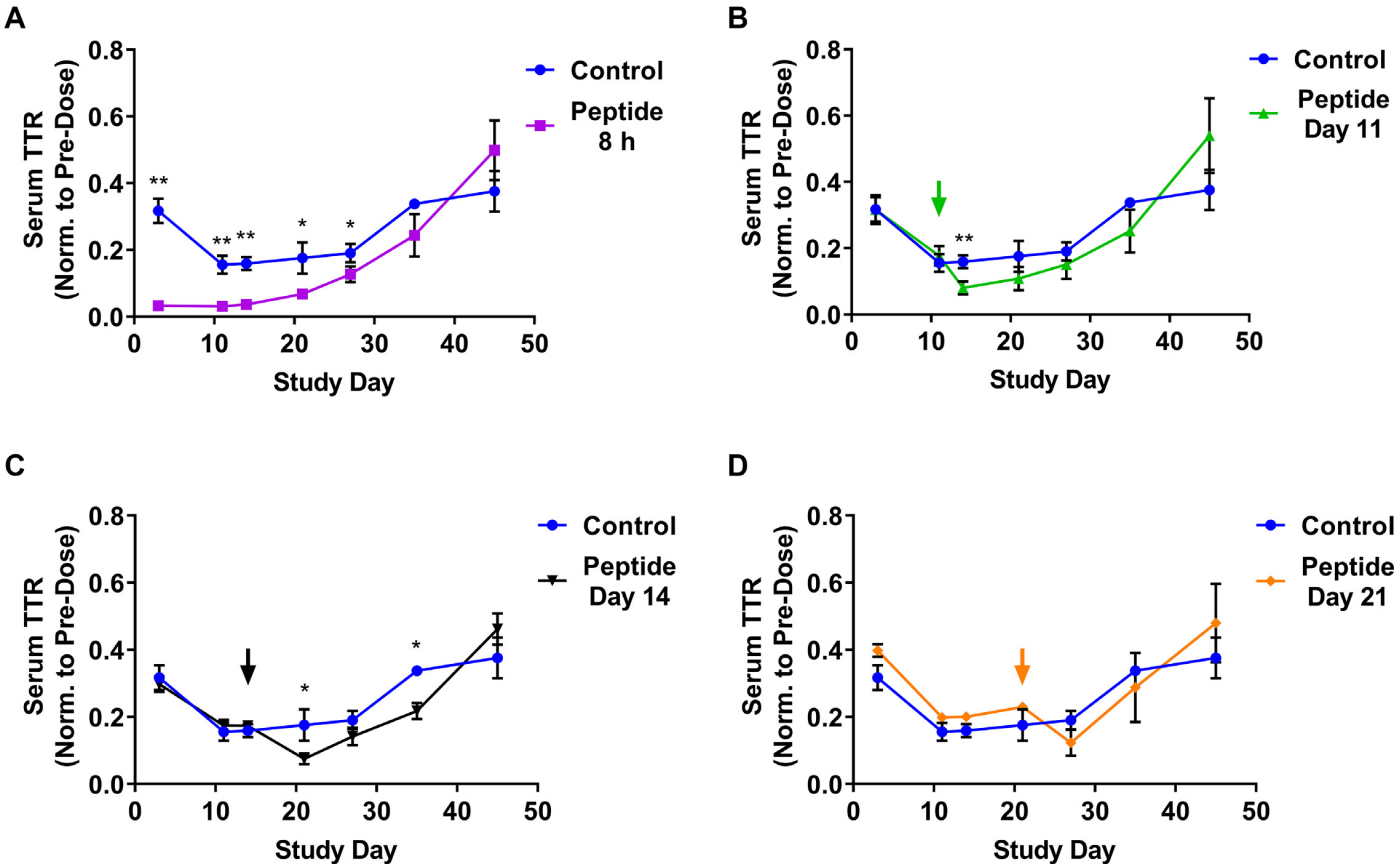
Chemically stabilized siRNA survives for weeks in acidic intracellular compartments. An Advanced ESC GalNAc–siRNA (siTTR-2) targeting *Ttr* was dosed SC at 0.5 mg/kg (all cohorts including the control group, which is shown in each graph as a comparison). GalNAc-INF7 endolytic peptide was dosed SC at 5 mg/kg in cohorts of mice at either 8 h (**A**) or days 11 (**B**), 14 (**C**) or 21 (**D**) post siRNA dose. Serum TTR protein levels were monitored through day 45. Data is represented as mean ± SD, for *N* = 3. Welch's independent t-test was utilized to compare the mean value between the control and peptide-treated groups at each timepoint to identify significant differences in target knockdown(*P ≤ 0.05, **P ≤ 0.01). Full results for the statistical analysis are included in the Supplemental Information.

Next, we examined the stability of Advanced ESC siRNA over time by administering GalNAc-INF7 peptide 8 hours, and 11, 14- and 21-days post siRNA dose (0.5 mg/kg, Figure 8). Functional siRNA could be released from acidic compartments through day 21, leading to enhanced knockdown after each peptide administration (Figure 8). This observation supports the hypothesis that siRNA stability within the endolysosomal pathway is a key driver of the extended duration of activity observed *in vivo*.

**Figure 9.**
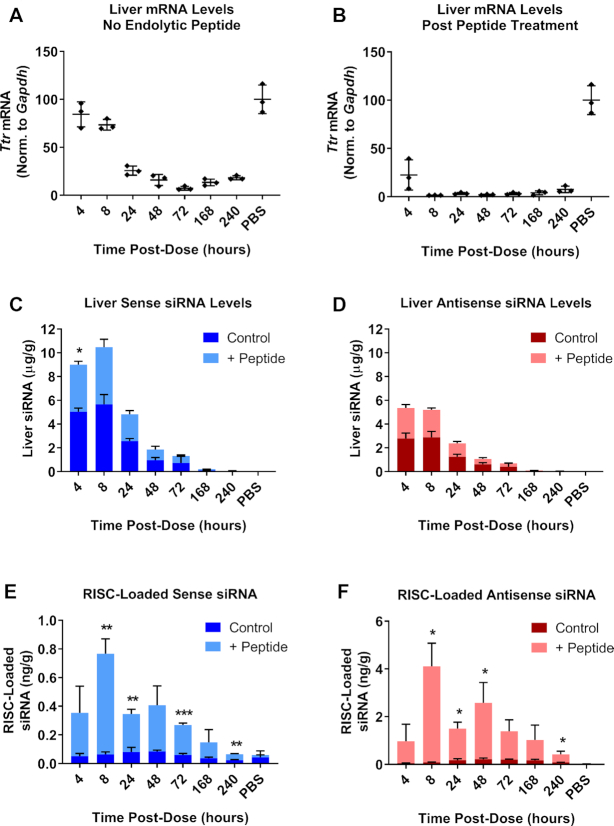
A large amount of functional siRNA is released and loaded into RISC following treatment with a GalNAc-conjugated endolytic peptide. An ESC GalNAc–siRNA (siTTR-1) targeting *Ttr* was dosed SC at 0.5 mg/kg. Half of the mice in the study were treated with 5 mg/kg of the GalNAc-INF7 endolytic peptide dosed SC 15 min after the siRNA dose. Cohorts of mice were sacrificed at several times post-dose (4 h through day 10). *Ttr* mRNA levels were quantified and normalized to *Gapdh* in control animals (**A**) or those that were treated with GalNAc-INF7 peptide (**B**). (**C**) Total siRNA sense strand liver levels were quantified by RT-qPCR in control (dark blue) or endolytic peptide-treated animals (light blue). (**D**) Total siRNA antisense strand liver levels were quantified by RT-qPCR in control (dark red) or endolytic peptide-treated animals (light red). (**E**) Sense strand RISC loading was quantified by RT-qPCR in control (dark blue) or endolytic peptide-treated animals (light blue). (**F**) Antisense strand RISC loading was quantified by RT-qPCR in control (dark red) or endolytic peptide-treated animals (light red). Additional control pull downs were performed with a mutant APP peptide (Supplementary Figure S3). Data is represented as mean ± SD, for *N* = 3. Welch's independent t-test was utilized to compare the mean value between the control and peptide-treated groups at each timepoint to identify significant differences in siRNA liver levels and RISC loading (*P ≤ 0.05, **P ≤ 0.01, ***P ≤ 0.001). Full results for the statistical analysis are included in the Supplemental Information.

To further interrogate the underlying biology of the enhanced siRNA activity following endolytic peptide administration, we evaluated *Ttr* knockdown, total siRNA liver levels and RISC loading at early time points (four hours through day 10). We treated mice with an ESC conjugate targeting *Ttr* at 1.5 mg/kg (Figure 9). Half of the mice were then treated with 5 mg/kg GalNAc-INF7 15 min post siRNA dose. Administration of GalNAc–INF7 led to a dramatic increase in both degree and onset of knockdown, with maximum knockdown occurring after only 8 h (∼98%) as opposed to the control cohort (without GalNAc-INF7 administration) demonstrating maximum knockdown on day 3 (∼93%) (Figure 9A, B). GalNAc-INF7 also increased the duration of the enhanced knockdown, maintaining 93% KD through day 10, while control mice had already recovered to 73% KD by day 10. Total siRNA liver level analysis revealed no significant differences between the cohorts, suggesting that peptide administration did not affect total siRNA levels in the liver (Figure 9C, D). Therefore, the amount of siRNA released from intracellular acidic compartments constitutes a small fraction of the total siRNA in the liver. Finally, we measured RISC loading for all cohorts and found a striking difference in groups treated with peptide versus the GalNAc–siRNA alone control groups. While the peak of RISC loading and greatest knockdown occurred after 2–3 days in the control animals, RISC loading was highest at 8 h in the peptide treated animals, consistent with the highest knockdown in these groups. At its peak, RISC loading in the peptide group was 20-fold higher (8 h) than the peak RISC loading in the control groups (48–72 h) suggesting there is substantial additional capacity for RISC loading in hepatocytes (Figure 9E, F). This very high level of RISC loading may contribute to the extended duration seen in this experiment due to the half-life of loaded RISC determined previously (3.8 days, Figure 5) and is unlikely due to the intracellular depot. However, even with the assistance of the endosomal release peptide, the total concentration of RISC-loaded antisense strand at 8 hours was 4 ng/g, which represents only 0.17% of the total antisense strand present in the liver at that time. The observation that even a small increase in cytosolic siRNA can greatly increase target knockdown illustrates that endolysosomal release is a critical rate-limiting step in the delivery of GalNAc–siRNA.

### Newly translated Ago2 loads siRNA several weeks after dosing

**Figure 10.**
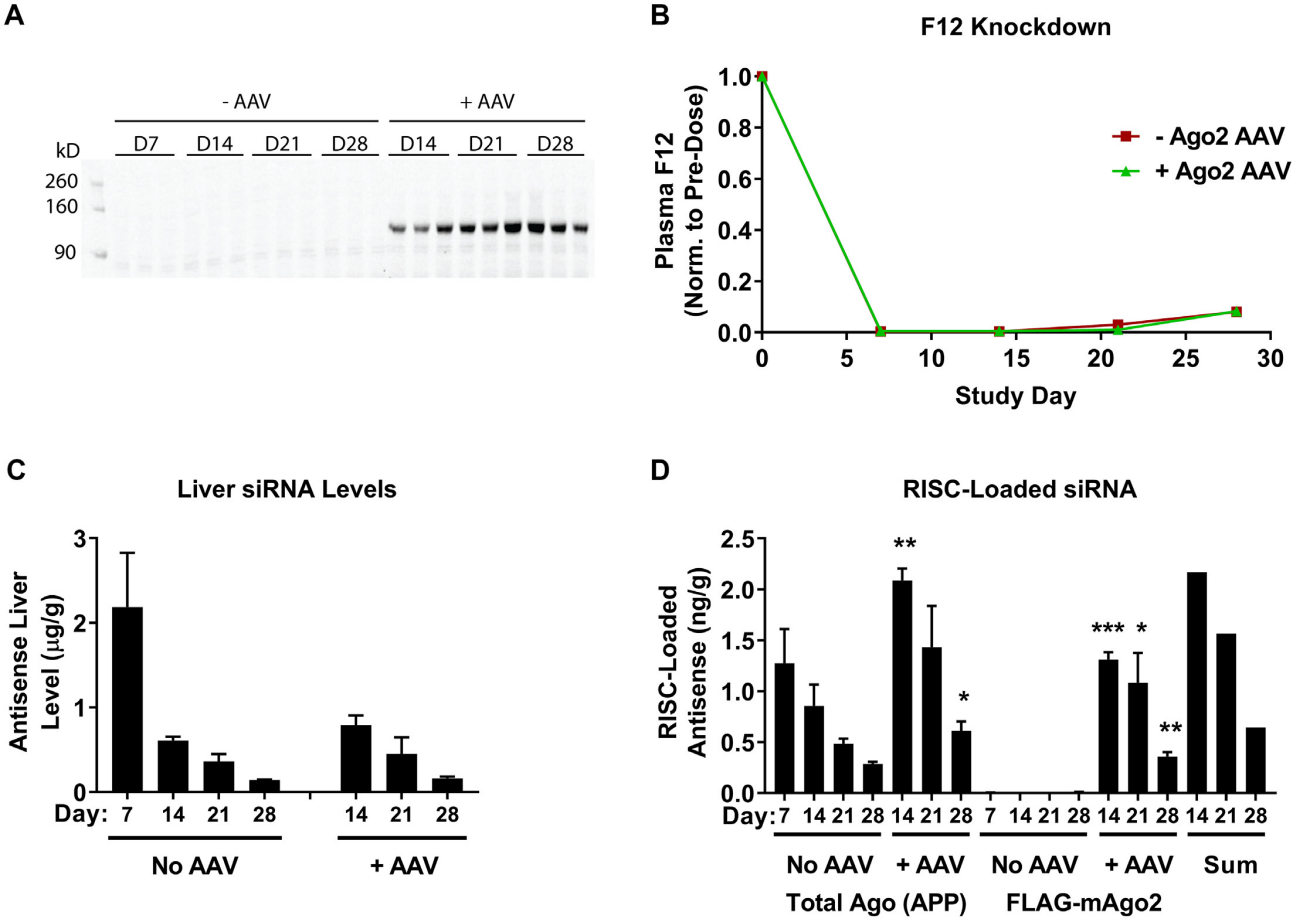
Exogenously expressed Ago2 loads F12 siRNA (siF12-1, 3 mg/kg) 1–3 weeks after dosing. An AAV vector was designed to express 3xFLAG-tagged mouse Ago2 (FLAG-mAgo2) under a constitutive liver-specific promoter (TBG). (**A**) Expression of the FLAG-mAgo2 protein was verified in AAV treated mice by Western Blot using an anti-FLAG antibody. Days refer to days post siRNA dose (day 0). FLAG-mAgo2 AAV was injected into three cohorts of mice on day 7 of the experiment (**B**) Plasma F12 protein levels were quantified by ELISA and normalized to pre-dose for each animal. (**C**) F12 antisense siRNA liver levels were quantified by RT-qPCR. (**D**) RISC loading was performed by anti-FLAG IP for both -AAV and +AAV groups (FLAG-mAgo2) and by Ago-APP for -AAV and +AAV groups (Total Ago (APP)). Loaded antisense siRNA was quantified by RT-qPCR. Data is represented as mean ± SD, for N = 3. Welch's independent t-test was utilized to compare the mean value between the control and +AAV groups at each timepoint in (C, D), with statistically significant increases in either liver level or RISC loading shown for the +AAV groups (*P ≤ 0.05, **P ≤ 0.01, ***P ≤ 0.001). Full results for the statistical analysis are included in the Supplemental Information.

Our data suggest that the half-life of loaded Ago2 cannot support the extended duration of effect seen with GalNAc–siRNA conjugates (Figures 3–5). Instead, the finding that chemically stabilized GalNAc–siRNAs reside in acidic compartments supports a model in which siRNA is slowly released to the cytosol to support continual loading into RISC, preserving target knockdown over time. To investigate this possibility, we expressed a 3xFLAG-tagged mouse Ago2 (FLAG-mAgo2) from an AAV vector under a constitutively active, liver-specific promoter (TBG). Several groups of mice were dosed on day 0 at 3 mg/kg with a *F12*-targeting Advanced ESC siRNA (siF12-1), and some groups were injected with FLAG-mAgo2 AAV seven days later (day 7). Cohorts of animals with and without AAV were sacrificed over a period of three weeks after the day 7 AAV injections and liver expression of FLAG-mAgo2 was confirmed on days 14, 21 and 28 (Figure 10A). We observed that total siRNA liver levels were similar in animals with or without AAV and that *F12* knockdown was saturated through day 21 post siRNA dose (Figure 10B-C). Quantification of Ago2-loaded siRNA was performed using two pull down approaches (Figure 10D). For total Argonaute protein pull down we performed peptide-based affinity purification (Ago-APP), which pulls down all endogenous Argonaute proteins including the AAV-expressed FLAG-mAgo2 (32,33). To selectively pull down AAV-expressed FLAG-mAgo2 we used an anti-FLAG antibody. Animals that were not injected with AAV did not show any siRNA signal in the FLAG antibody pull downs (Figure 10D). However, animals dosed with AAV showed significant siRNA signal in the FLAG antibody pull downs. Interestingly, the total amount of Ago-loaded antisense strand pulled down by Ago-APP in the +AAV cohort was roughly equivalent to the sum of total antisense strand pulled down in animals that were not dosed with AAV plus the amount pulled down by the FLAG antibody in the +AAV cohort (Figure 10D, ‘Sum’ bars). Thus, siRNA can be loaded into newly translated Ago2 1–3 weeks after dosing. Coupled with the Ago2 half-life calculation of ∼3.8 days, these results suggest that siRNA is continuously loaded into Ago2 over time and that the supply of siRNA in the cytosol can sustain persistent RNAi activity.

## DISCUSSION

Advances in siRNA design, primarily through chemical modification for increased metabolic stability, have led to steady improvements in potency and duration of GalNAc–siRNA conjugates (9,12). Clinical trials evaluating the safety and efficacy of investigational RNAi therapeutics show that infrequent dosing paradigms can support robust and sustained target knockdown; data from the ORION-11 clinical trial that evaluated a GalNAc–siRNA targeting PCSK9 support a biannual dosing regimen to maintain >50% lowering of LDL cholesterol (45). We investigated the biological origins of this extended duration of activity by performing *in vitro* and *in vivo* studies in rodents to interrogate multiple possibilities, including slow release of compound from the injection site, half-life of functional RISC and an intracellular depot sustaining the formation of functional RISC for extended periods of time. Overall, we find increased half-life of chemically stabilized siRNA in acidic intracellular compartments, such as lysosomes, is the predominant driver for the extended duration of activity. We propose that a slow release of stabilized siRNA from acidic compartments enables continuous loading of RISC and prolonged target silencing. The duration of activity correlates well with the metabolic stability of the siRNA. Less stable siRNA designs degrade faster in the acidic compartments and are therefore unable to support continuous RISC loading over time. Indeed, Advanced ESC siRNAs outperform earlier ESC siRNAs in mice and non-human primates (NHP), leading to higher total siRNA liver levels, increased loading into RISC, and an extended duration of target knockdown (12). One significant difference between ESC and Advanced ESC designs is the increased 2′-OMe content of Advanced ESC siRNAs. This bulky modification has been shown to increase the nuclease resistance and metabolic stability of oligonucleotides compared to less bulky modifications like 2′-F (46,47). However, careful placement of modifications is critical to balance retaining inherent RNAi activity while maximizing metabolic stability. Indeed, we previously showed that a fully 2′-OMe siRNA, which was the most stable design as measured by total siRNA liver levels, was unable to load into RISC and failed to knock down its target mRNA (12). Thus, although siRNA stability drives improvements in knockdown and duration, potency benefits can be lost if the template design is not amenable to RISC loading.

It is well established that the natural trafficking pathway of ligand bound ASGPR relies on endosomal maturation and acidification to dissociate the receptor from its cargo. The liberated cargo is then degraded in lysosomes while ASGPR recycles to the cell surface (48–51). Thus, siRNA cargo is likely exposed to acidic conditions from very early stages in the trafficking pathway. We have shown that siRNA is localized to acidic intracellular compartments long after dosing and that the extended duration of RNAi activity is directly related to chemical stability. However, we cannot rule out the possibility of more than one site of siRNA accumulation in the cell. Other compartments, such as the cytosol, could play a role in durability. Stabilization of the antisense strand in the RISC complex only appears to play a minor role for the observed duration of activity, given the limited ∼3.8 day half-life in mice. miRNAs have also been shown to be more stable and resistant to degradation once loaded into the RISC complex with half-lives in the same range as the antisense strand tested herein (52,53).

Both the precise identity of the acidic intracellular compartments where siRNA accumulates and how exactly the siRNA slowly escape are open questions. The storage compartments could be late endosomes, lysosomes or multivesicular bodies (MVB), which are all acidic and dynamic bodies undergoing constant changes as they interact with other intracellular compartments. Sequestration of siRNA in endolysosomal compartments is a major rate-limiting step for siRNA delivery to the cytosol and subsequent RISC loading. Methods to overcome this entrapment using various agents has been extensively evaluated and include: osmolytic agents such as nigericin and chloroquine to induce vesicle swelling and bursting, cell penetrating and fusogenic peptides such as EB1, melittin and dfTAT to disrupt membrane integrity similar to the INF7 peptide variant used herein, photochemical stimulation to induce compound internalization, and inhibition of endogenous proteins involved in membrane repair to make vesicles more permeable (18–20,54). These methods can increase functional siRNA release; however, they are not practical therapeutic approaches due to unacceptable toxicity. The exact nature of siRNA release is unclear but components of the ESCRT machinery that mediate budding into the endomembrane lumen appear to be important for siRNA activity as they cause transient perturbations of local membrane structure that facilitate siRNA escape (16,55,56). Another factor that contributes to oligonucleotide activity is the formation of intraluminal vesicles (ILVs) within multi-vesicular bodies (MVBs) that back-fuse to the membrane, a process that can release ASOs into the cytosol (57). Importantly, RISC has been shown to be associated with late endosome membranes, inside ILVs and on the cytoplasmic face of the rough ER, placing it within close proximity to these putative sites of escape into the cytosol (58–61). Elucidating the mechanism of siRNA release from defined intracellular depots is the subject of ongoing and future work.

## DATA AVAILABILITY

The authors declare that all data supporting the findings of this study are available within the article and the Supplementary Information files.

## Supplementary Material

gkaa670_Supplemental_File
