# Supplementary material for: Investigating the pharmacodynamic durability of GalNAc–siRNA conjugates
Source: Nucleic Acids Res. 2020 Aug 18;48(21):11827–44. doi: 10.1093/nar/gkaa670 (PMC7708070; doi:10.1093/nar/gkaa670)
Supplement: gkaa670_Supplemental_File [file gkaa670_supplemental_file.pdf]

Supplementary Information:

Investigating the Pharmacodynamic Durability of GalNAc-siRNA Conjugates

Christopher R. Brown[1], Swati Gupta[1], June Qin[1], Timothy Racie[1], Guo He[1], Scott Lentini[1], Ryan Malone[1], Mikyung Yu[1], Shigeo Matsuda[1], Svetlana Shulga-Morskaya[1], Anil V. Nair[2], Christopher S. Theile[1], Karyn Schmidt[1], Azar Shahraz[1], Varun Goel[1], Rubina G. Parmar[1], Ivan Zlatev[1], Mark K. Schlegel[1], Jayaprakash K. Nair[1], Muthusamy Jayaraman[1], Muthiah Manoharan[1], Dennis Brown[2], Martin A. Maier[1], Vasant Jadhav[1*]

[1]Alnylam Pharmaceuticals, Inc., Cambridge, MA 02142, USA

[2]MGH Program in Membrane Biology, Division of Nephrology, Massachusetts General Hospital and Harvard Medical School, Boston, MA 02114, USA

[*] Correspondence should be addressed to V.J. (vjadhav@alnylam.com)

Table of Contents

Supplementary Methods.. 3

Compound **802** MALDI data file. 3

Compound **804** MALDI data file. 4

Statistical Supplemental Methods for Figure 7 5

Statistical Supplemental Methods for Figure 8 9

Statistical Supplemental Methods for Figure 9 13

Statistical Supplemental Methods for Figure 10 17

Supplemental Table S1 19

Supplementary Figures 20

Supplementary Reference.. 22

SUPPLEMENTARY METHODS

Compound **802** MALDI data file.

Mass calc. for $C_{75}H_{128}N_{12}O_{29}S_2$: 1726.02, found: 1750.084 (M+Na) MALDI.

Comment 1
Comment 2

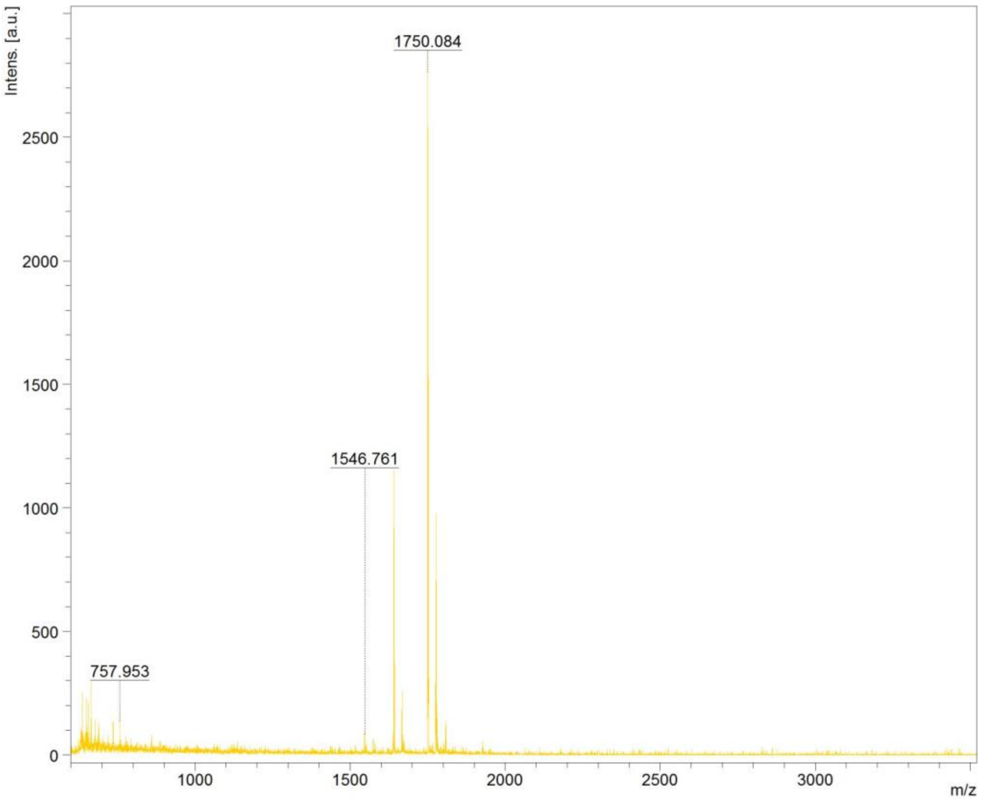

Acquisition Parameter

Date of acquisition	2020-06-10T10:35:47.268-04:00
Acquisition method name	D:\Methods\flexControlMethods\RP_1000-3000_Da.par
Aquisition operation mode	Reflector
Voltage polarity	POS
Number of shots	500
Name of spectrum used for calibration	
Calibration reference list used	PeptideCalibStandard mono

Instrument Info

User	joy
Instrument	FLEX-PC
Instrument type	microflex

Compound **804** MALDI data file.

Mass calc. for $C_{189}H_{303}N_{39}O_{70}S_2$: 4305.83, found: 4329.858 (M+Na) MALDI.

D:\Data\Specs\2016Joy\2018-Ashish\20180607\RP-998-156\0_F10\1\1Ref

Comment 1

Comment 2

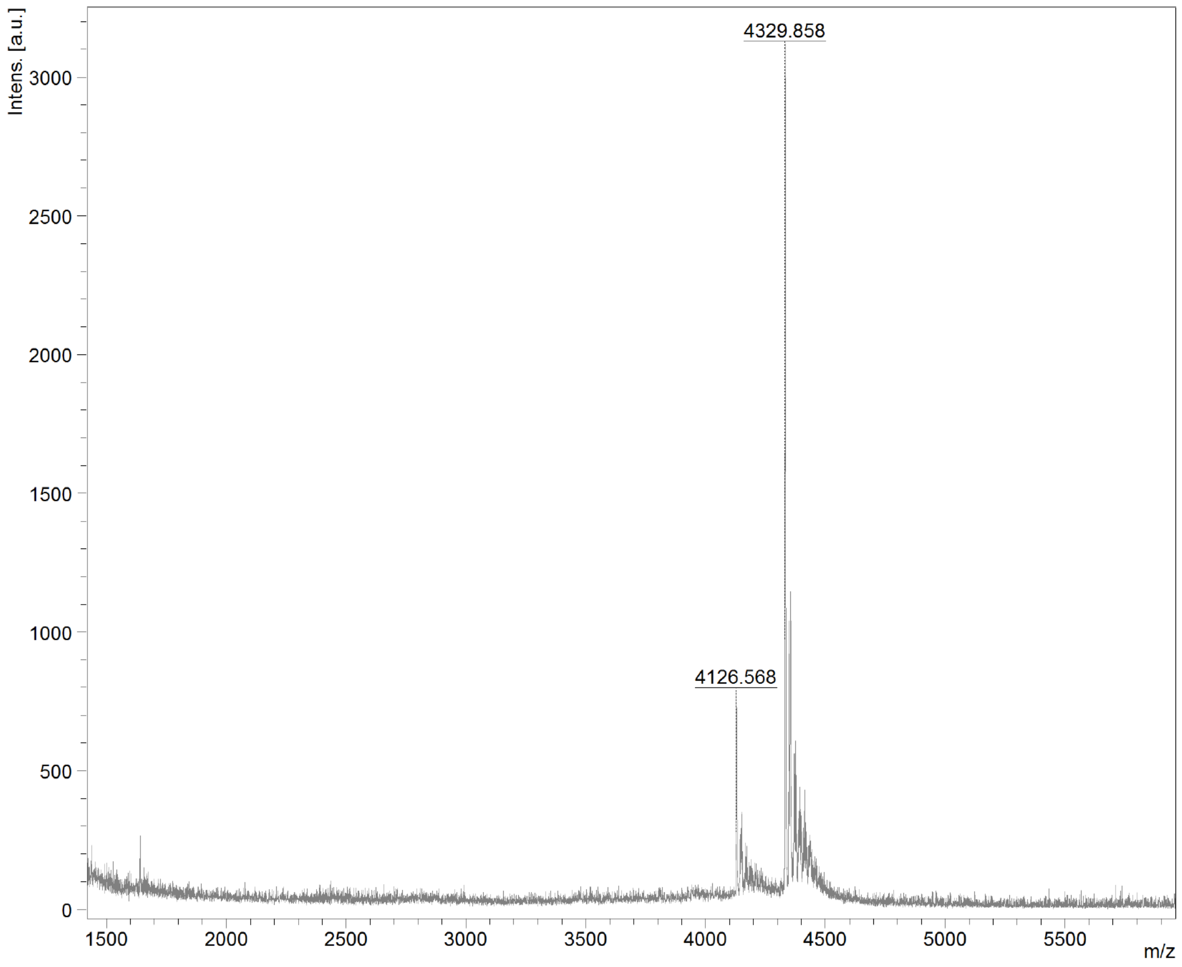

Acquisition Parameter

Date of acquisition	2019-02-06T07:52:40.736-05:00
Acquisition method name	D:\Methods\flexControlMethods\RP_1000-3000_Da.par
Aquisition operation mode	Reflector
Voltage polarity	POS
Number of shots	1107
Name of spectrum used for calibration	
Calibration reference list used	PeptideCalibStandard mono

Statistical Supplemental Methods for Figure 7
Method of analysis:

Due to unequal variance, Welch's independent t-test was utilized to compare the mean value between the control and peptide-treated group at each timepoint.

Statistics for Figure 7A - Comparison of serum TTR levels between control and treatment group where peptide was administered at day 7 following ESC siRNA (1.5mg/kg, siTTR-1).

Timepoint (days)		*Control*	*+ Peptide*	*Difference*	*95% CI Upper*	*95% CI Lower*	*t*	*df*	*p-value*
	M	0.24	0.23						
3	*±SD*	0.03	0.05	-0.01	0.10	-0.11	-0.25	2.98	0.82
	N	3	3						
	M	0.25	0.21						
7	*±SD*	0.09	0.04	-0.04	0.16	-0.23	-0.67	2.87	0.55
	N	3	3						
	M	0.23	0.17						
10	*±SD*	0.02	0.07	-0.05	0.09	-0.20	-1.38	2.34	0.28
	N	3	3						
	M	0.33	0.27						
14	*±SD*	0.06	0.08	-0.06	0.11	-0.23	-0.98	3.49	0.39
	N	3	3						
	M	0.55	0.49						
21	*±SD*	0.09	0.13	-0.06	0.19	-0.32	-0.71	3.66	0.52
	N	3	3						
	M	0.92	0.87						
35	*±SD*	0.06	0.13	-0.05	0.23	-0.34	-0.62	2.87	0.58
	N	3	3						

All p-values are derived from a two-tailed hypothesis test. M = mean, SD = standard deviation, N = number of animals.

Statistical Supplement for Figure 7

Statistics for Figure 7B - Comparison of serum TTR levels between control and treatment group where peptide was administered at day 7 following Advanced ESC siRNA (0.5mg/kg, siTTR-3).

Timepoint (days)		*Control*	*+Peptide*	*Difference*	*95% CI Upper*	*95% CI Lower*	*t*	*df*	*p-value*
	M	0.44	0.57						
3	*±SD*	0.07	0.08	0.13	0.29	-0.03	2.23	3.93	0.09
	N	3	3						
	M	0.25	0.32						
7	*±SD*	0.02	0.03	0.07	0.13	0.01	3.49	3.18	0.04
	N	3	3						
	M	0.26	0.10						
10	*±SD*	0.04	0.03	-0.16	-0.08	-0.24	-5.83	3.36	0.007
	N	3	3						
	M	0.25	0.10						
14	*±SD*	0.08	0.05	-0.15	0.02	-0.32	-2.65	3.20	0.072
	N	3	3						
	M	0.33	0.20						
21	*±SD*	0.08	0.03	-0.13	0.35	-0.60	-2.18	1.24	0.23
	N	2	2						
	M	0.53	0.38						
35	*±SD*	0.12	0.05	-0.15	0.54	-0.84	-1.59	1.31	0.31
	N	2	2						

All p-values are derived from a two-tailed hypothesis test. M = mean, SD = standard deviation, N = number of animals.

Statistical Supplement for Figure 7

Statistics for Figure 7C - Comparison of serum TTR levels between control and treatment group where peptide was administered 15 minutes following ESC siRNA (1.5mg/kg, siTTR-1).

Timepoint (days)		*Control*	*+ Peptide*	*Difference*	*95% CI Upper*	*95% CI Lower*	*t*	*df*	*p-value*
	M	0.24	0.04						
3	*±SD*	0.03	0.01	-0.20	-0.13	-0.26	-12.10	2.23	0.004
	N	3	3						
	M	0.25	0.02						
7	*±SD*	0.09	0.01	-0.23	-0.00	-0.46	-4.27	2.02	0.05
	N	3	3						
	M	0.23	0.02						
10	*±SD*	0.02	0.02	-0.21	-0.16	-0.26	-12.62	3.92	0.0003
	N	3	3						
	M	0.33	0.03						
14	*±SD*	0.06	0.01	-0.30	-0.17	-0.43	-9.25	2.14	0.009
	N	3	2						
	M	0.55	0.20						
21	*±SD*	0.09	0.14	-0.35	-0.07	-0.63	-3.65	3.51	0.03
	N	3	3						
	M	0.92	0.52						
35	*±SD*	0.06	0.13	-0.40	-0.13	-0.68	-4.72	2.90	0.02
	N	3	3						

All p-values are derived from a two-tailed hypothesis test. M = mean, SD = standard deviation, N = number of animals.

Statistical Supplement to Figure 7

Statistics for Figure 7D - Comparison of serum TTR levels between control and treatment group where peptide was administered 15 minutes following ESC siRNA (0.5mg/kg, siTTR-1).

Timepoint (days)		*Control*	*+ Peptide*	*Difference*	*95% CI Upper*	*95% CI Lower*	*t*	*df*	*p-value*
	M	0.59	0.12						
3	*+SD*	0.12	0.07	-0.48	-0.22	-0.73	-5.76	3.27	0.008
	N	3	3						
	M	0.61	0.14						
7	*+SD*	0.03	0.10	-0.47	-0.24	-0.69	-7.77	2.35	0.01
	N	3	3						
	M	0.66	0.18						
10	*+SD*	0.02	0.12	-0.48	-0.19	-0.76	-6.82	2.12	0.02
	N	3	3						
	M	0.80	0.33						
14	*+SD*	0.10	0.18	-0.47	-0.10	-0.84	-3.95	3.16	0.03
	N	3	3						
	M	1.10	0.65						
21	*+SD*	0.08	0.21	-0.44	0.01	-0.90	-3.42	2.58	0.053
	N	3	3						
	M	1.07	1.26						
35	*+SD*	0.15	0.29	0.19	0.79	-0.40	1.03	3.00	0.38
	N	3	3						

All p-values are derived from a two-tailed hypothesis test. M = mean, SD = standard deviation, N = number of animals.

Statistical Supplemental Methods for Figure 8

Method of analysis:

Due to unequal variance, Welch's independent t-test was utilized to compare the mean value between the control and peptide-treated group at each timepoint.

Statistics for Figure 8A - Comparison of Serum TTR levels between control and treatment group where peptide was administered at 8 hours following Advanced ESC GalNAc-siRNA (0.5 mg/kg, siTTR-2).

Timepoint (days)		*Control*	*+ Peptide*	*Difference*	*95% CI Upper*	*95% CI Lower*	*t*	*df*	*p-value*
	M	31.72	3.27						
3	*±SD*	3.69	0.73	-28.5	-19.2	-37.2	-13.1	2.15	0.004
	N	3	3						
	M	15.55	3.14						
11	*±SD*	2.67	1.42	-12.4	-6.9	-17.9	-7.1	3.05	0.005
	N	3	3						
	M	15.91	3.65						
14	*±SD*	1.97	1.30	-12.3	-8.2	-16.3	-8.9	3.46	0.002
	N	3	3						
	M	17.60	6.77						
21	*±SD*	3.62	2.32	-10.8	-3.4	-18.2	-4.4	3.41	0.02
	N	3	3						
	M	19.01	12.73						
27	*±SD*	2.78	2.37	-6.3	-0.4	-12.2	-2.9	3.9	0.04
	N	3	3						
	M	33.80	24.37						
35	*±SD*	0.12	6.31	-9.4	6.2	-25.1	-2.6	2.0	0.12
	N	2	3						
	M	37.60	49.83						
45	*±SD*	6.12	8.96	12.2	30.6	-6.1	1.95	3.53	0.13
	N	3	3						

All p-values are derived from a two-tailed hypothesis test. M = mean, SD = standard deviation, N = number of animals.

Statistical Supplement to Figure 8

Statistics for Figure 8B - Comparison of serum TTR levels between control and treatment group where peptide was administered at day 11 following Advanced ESC GalNAc-siRNA (0.5 mg/kg, siTTR-2).

Timepoint (days)		*Control*	*+ Peptide*	*Difference*	*95% CI Upper*	*95% CI Lower*	*t*	*df*	*p-value*
	M	31.72	31.61						
3	*+SD*	3.69	4.39	-0.1	9.19	-9.42	-0.03	3.88	0.974
	N	3	3						
	M	15.55	17.71						
11	*+SD*	2.67	2.93	2.2	8.54	-4.22	0.94	3.97	0.400
	N	3	3						
	M	15.91	8.04						
14	*+SD*	1.97	1.93	-7.9	-3.45	-12.31	-4.94	3.99	0.008
	N	3	3						
	M	17.60	10.83						
21	*+SD*	3.62	2.83	-6.8	0.76	-14.31	-2.56	3.78	0.066
	N	3	3						
	M	19.01	15.09						
27	*+SD*	2.78	4.41	-3.9	5.10	-12.93	-1.3	3.37	0.28
	N	3	3						
	M	33.80	25.17						
35	*+SD*	0.12	6.49	-8.6	7.48	-24.75	-2.3	2.00	0.15
	N	2	3						
	M	37.60	53.94						
45	*+SD*	6.12	11.30	16.3	39.61	-6.93	2.2	3.08	0.11
	N	3	3						

All p-values are derived from a two-tailed hypothesis test. M = mean, SD = standard deviation, N = number of animals.

Statistical Supplement to Figure 8

Statistics for Figure 8C - Comparison of serum TTR levels between control and treatment group where peptide was administered at day 14 following Advanced ESC GalNAc-siRNA (0.5 mg/kg, siTTR-2).

Timepoint (days)		*Control*	*+ Peptide*	*Difference*	*95% CI Upper*	*95% CI Lower*	*t*	*df*	*p-value*
	M	31.72	29.79						
3	*±SD*	3.69	2.29	-1.93	5.61	-9.47	-0.77	3.34	0.49
	N	3	3						
	M	15.55	17.46						
11	*±SD*	2.67	1.68	1.90	7.36	-3.55	1.04	3.37	0.37
	N	3	3						
	M	15.91	17.36						
14	*±SD*	1.97	0.77	1.45	5.71	-2.80	1.19	2.60	0.33
	N	3	3						
	M	17.60	7.51						
21	*±SD*	3.62	0.77	-10.09	-1.60	-18.58	-4.72	2.18	0.04
	N	3	3						
	M	19.01	14.14						
27	*±SD*	2.78	2.64	-4.87	1.29	-11.02	-2.20	3.99	0.093
	N	3	3						
	M	33.80	21.79						
35	*±SD*	0.12	2.40	-12.02	-6.08	-17.96	-8.65	2.01	0.01
	N	2	3						
	M	37.60	46.19						
45	*±SD*	6.12	4.71	8.59	21.30	-4.12	1.93	3.75	0.13
	N	3	3						

All p-values are derived from a two-tailed hypothesis test. M = mean, SD = standard deviation, N = number of animals.

Statistical Supplement to Figure 8

Statistics for Figure 8D - Comparison of serum TTR levels between control and treatment group where peptide was administered at day 21 following Advanced ESC GalNAc-siRNA (0.5 mg/kg, siTTR-2).

Timepoint (days)		*Control*	*+ Peptide*	*Difference*	*95% CI Upper*	*95% CI Lower*	*t*	*df*	*p-value*
3	*M*	31.72	36.50	4.78	16.70	-7.15	1.20	3.38	0.31
	+SD	3.69	5.84						
	N	3	3						
11	*M*	15.55	14.30	-1.26	20.76	-23.27	-0.22	2.30	0.85
	+SD	2.67	9.67						
	N	3	3						
14	*M*	15.91	14.62	-1.29	20.67	-23.25	-0.23	2.18	0.84
	+SD	1.97	9.34						
	N	3	3						
21	*M*	17.60	17.83	0.23	19.35	-18.90	0.04	2.64	0.97
	+SD	3.62	8.92						
	N	3	3						
27	*M*	19.01	12.27	-6.74	1.26	-14.74	-2.44	3.62	0.078
	+SD	2.78	3.90						
	N	3	3						
35	*M*	33.80	28.77	-5.04	87.25	-97.33	-0.69	1.00	0.61
	+SD	0.12	10.28						
	N	2	2						
45	*M*	37.60	47.97	10.37	71.39	-50.64	1.16	1.38	0.41
	+SD	6.12	11.64						
	N	3	2						

All p-values are derived from a two-tailed hypothesis test. M = mean, SD = standard deviation, N = number of animals.

Statistical Supplemental Methods for Figure 9
Method of analysis:

Due to unequal variance, Welch's independent t-test was utilized to compare the mean value between the control and peptide-treated group at each timepoint.

Statistics for Figure 9C - Comparison of sense strand siRNA liver levels between control and treatment group where peptide was administered 15 minutes following ESC GalNAc-siRNA (0.5 mg/kg, siTTR-1).

Timepoint (hours)		*Control*	*+ Peptide*	*Difference*	*95% CI Upper*	*95% CI Lower*	*t*	*df*	*p-value*
	M	5.034	3.97						
4	*±SD*	0.31	0.28	1.07	1.74	0.39	4.37	3.96	0.012
	N	3	3						
	M	5.67	4.81						
8	*±SD*	0.81	0.65	0.86	2.56	-0.85	1.43	3.81	0.23
	N	3	3						
	M	2.56	2.27						
24	*±SD*	0.22	0.30	0.29	0.92	-0.33	1.36	3.65	0.25
	N	3	3						
	M	0.97	0.89						
48	*±SD*	0.22	0.28	0.077	0.65	-0.50	0.38	3.79	0.72
	N	3	3						
	M	0.74	0.58						
72	*±SD*	0.56	0.087	0.16	1.50	-1.18	0.49	2.10	0.67
	N	3	3						
	M	0.09	0.12						
168	*±SD*	0.023	0.006	-0.032	0.022	-0.085	-2.30	2.27	0.13
	N	3	3						
	M	0.032	0.034						
240	*±SD*	0.0065	0.0043	-0.002	0.011	-0.015	-0.45	3.49	0.68
	N	3	3						

All p-values are derived from a two-tailed hypothesis test. M = mean, SD = standard deviation, N = number of animals.

Statistical Supplement to Figure 9

Statistics for Figure 9D - Comparison of antisense strand siRNA liver levels between control and treatment group where peptide was administered 15 minutes following ESC GalNAc-siRNA (0.5 mg/kg, siTTR-1).

Timepoint (hours)		*Control*	*+ Peptide*	*Difference*	*95% CI Upper*	*95% CI Lower*	*t*	*df*	*p-value*
	M	2.80	2.55						
4	*±SD*	0.45	0.30	-0.24	0.68	-1.16	-0.77	3.47	0.49
	N	3	3						
	M	2.89	2.32						
8	*±SD*	0.50	0.15	-0.57	0.55	-1.69	-1.90	2.35	0.18
	N	3	3						
	M	1.24	1.14						
24	*±SD*	0.22	0.16	-0.099	0.36	-0.56	-0.63	3.57	0.57
	N	3	3						
	M	0.62	0.44						
48	*±SD*	0.13	0.11	-0.18	0.11	-0.46	-1.75	3.92	0.16
	N	3	3						
	M	0.41	0.29						
72	*±SD*	0.29	0.033	-0.12	0.60	-0.84	-0.70	2.05	0.55
	N	3	3						
	M	0.042	0.053						
168	*±SD*	0.013	0.01	0.011	0.038	-0.015	1.19	3.85	0.30
	N	3	3						
	M	0.015	0.015						
240	*±SD*	0.0031	0.0007	-0.00012	0.0072	-0.0074	-0.064	2.22	0.95
	N	3	3						

All p-values are derived from a two-tailed hypothesis test. M = mean, SD = standard deviation, N = number of animals.

Statistical Supplement to Figure 9

Statistics for Figure 9E - Comparison of sense strand RISC loading between control and treatment group where peptide was administered 15 minutes following ESC GalNAc-siRNA (0.5 mg/kg, siTTR-1).

Timepoint (hours)		*Control*	*+ Peptide*	*Difference*	*95% CI Upper*	*95% CI Lower*	*t*	*df*	*p-value*
4	*M*	0.052	0.302	-0.25	0.21	-0.71	-2.31	2.048	0.1448
	±SD	0.018	0.19						
	N	3	3						
8	*M*	0.063	0.703	-0.64	-0.39	-0.89	-10.48	2.12	0.0073
	±SD	0.018	0.10						
	N	3	3						
24	*M*	0.082	0.26	-0.18	-0.11	-0.26	-6.98	3.98	0.0023
	±SD	0.031	0.033						
	N	3	3						
48	*M*	0.084	0.32	-0.24	0.091	-0.57	-3.087	2.02	0.0897
	±SD	0.0094	0.13						
	N	3	3						
72	*M*	0.061	0.21	-0.15	-0.12	-0.18	-15.94	3.75	0.0001
	±SD	0.0099	0.013						
	N	3	3						
168	*M*	0.037	0.11	-0.075	0.14	-0.29	-1.46	2.034	0.2796
	±SD	0.0082	0.089						
	N	3	3						
240	*M*	0.023	0.042	-0.019	-0.009	-0.028	-5.54	3.9	0.0056
	±SD	0.0044	0.0038						
	N	3	3						

All p-values are derived from a two-tailed hypothesis test. M = mean, SD = standard deviation, N = number of animals.

Statistical Supplement to Figure 9

Statistics for Figure 9F - Comparison of antisense strand RISC loading between control and treatment group where peptide was administered 15 minutes following ESC GalNAc-siRNA (0.5 mg/kg, siTTR-1).

Timepoint (hours)		*Control*	*+ Peptide*	*Difference*	*95% CI Upper*	*95% CI Lower*	*t*	*df*	*p-value*
	M	0.05	0.92						
4	*+SD*	0.013	0.71	0.87	2.64	-0.91	2.1	2.0	0.17
	N	3	3						
	M	0.083	4.03						
8	*+SD*	0.013	0.97	3.94	6.35	1.54	7.06	2.0	0.02
	N	3	3						
	M	0.18	1.32						
24	*+SD*	0.06	0.27	1.14	1.77	0.51	7.13	2.2	0.015
	N	3	3						
	M	0.21	2.37						
48	*+SD*	0.054	0.85	2.15	4.26	0.045	4.36	2.02	0.048
	N	3	3						
	M	0.2	1.18						
72	*+SD*	0.02	0.48	0.98	2.18	-0.22	3.5	2.01	0.073
	N	3	3						
	M	0.17	0.86						
168	*+SD*	0.048	0.63	0.69	2.23	-0.85	1.9	2.023	0.20
	N	3	3						
	M	0.083	0.33						
240	*+SD*	0.011	0.14	0.25	0.59	-0.085	3.18	2.03	0.085
	N	3	3						

All p-values are derived from a two-tailed hypothesis test. M = mean, SD = standard deviation, N = number of animals.

Statistical Supplemental Methods for Figure 10

Method of analysis:

Due to unequal variance, Welch's independent t-test was utilized to compare the mean value of siRNA liver levels and RISC loading between the control and +AAV groups at the day 14, 21 and 28 timepoints.

Statistics for Figure 10C - Comparison of antisense strand siRNA liver levels between control (no AAV) and +AAV groups following Advanced ESC GalNAc-siRNA (3 mg/kg, siF12-1).

Timepoint (days)		*Control (ug/g)*	*+ AAV (ug/g)*	*Difference*	*95% CI Upper*	*95% CI Lower*	*t*	*df*	*p-value*
	M	0.60	0.79						
14	*±SD*	0.049	0.11	-0.19	0.056	-0.43	-2.6	2.72	0.089
	N	3	3						
	M	0.36	0.45						
21	*±SD*	0.087	0.19	-0.09	0.32	-0.5	-0.73	2.77	0.52
	N	3	3						
	M	0.14	0.16						
28	*±SD*	0.0063	0.02	-0.019	0.026	-0.064	-1.6	2.37	0.23
	N	3	3						

All p-values are derived from a two-tailed hypothesis test. M = mean, SD = standard deviation, N = number of animals.

Statistical Supplement to Figure 10

Statistics for Figure 10D – Ago-APP Comparison (data bars 2 through 7) - Comparison of antisense strand siRNA loaded into RISC between control (no AAV) and +AAV groups using the Ago-APP pulldown approach.

Timepoint (days)		*Control (ng/g)*	*+ AAV (ng/g)*	*Difference*	*95% CI Upper*	*95% CI Lower*	*t*	*df*	*p-value*
	M	0.86	2.09						
14	*±SD*	0.21	0.12	-1.23	-0.803	-1.66	-8.86	3.2	0.0024
	N	3	3						
	M	0.48	1.43						
21	*±SD*	0.05	0.41	-0.95	0.037	-1.93	-4.028	2.061	0.054
	N	3	3						
	M	0.29	0.61						
28	*±SD*	0.021	0.09	-0.33	-0.12	-0.53	-6.14	2.21	0.02
	N	3	3						

All p-values are derived from a two-tailed hypothesis test. M = mean, SD = standard deviation, N = number of animals.

Statistics for Figure 10D – FLAG-mAgo2 Comparison (data bars 9 through 14) - Comparison of antisense strand siRNA loaded into RISC between control (no AAV) and +AAV groups using the FLAG pulldown approach.

Timepoint (days)		*Control (ng/g)*	*+ AAV (ng/g)*	*Difference*	*95% CI Upper*	*95% CI Lower*	*t*	*df*	*p-value*
	M	0.003	1.31						
14	*±SD*	0.0002	0.073	-1.31	-1.13	-1.49	-32	2	0.001
	N	3	3						
	M	0.0038	1.082						
21	*±SD*	0.0001	0.29	-1.08	-0.35	-1.81	-6.34	2	0.024
	N	3	3						
	M	0.0088	0.36						
28	*±SD*	0.0041	0.045	-0.35	-0.24	-0.46	-13.51	2.03	0.0051
	N	3	3						

All p-values are derived from a two-tailed hypothesis test. M = mean, SD = standard deviation, N = number of animals.

SUPPLEMENTAL TABLE S1

RT-qPCR primers and probes for quantifying antisense strands.

siRNA	Primer	Sequence (5'-3')
siTTR	RT	GTCGTATCCAGTGCAGGGTCCGAGGTATTCGCACTGGATACGACAAAACAGTGT
	Forward qPCR	GCCGCGCTTATAGAGCAAG
	TaqMan Probe	CTGGATACGACAAAACAGT
	Reverse qPCR	GTGCAGGGTCCGAGGT
siF7	RT	GTCGTATCCAGTGCAGGGTCCGAGGTATTCGCACTGGATACGACGCCAGGATCA
	Forward qPCR	GCCGCGCTTAAGACTTGAGA
	TaqMan Probe	CTGGATACGACGCCAGGAT
	Reverse qPCR	GTGCAGGGTCCGAGGT
siF9	RT	GTCGTATCCAGTGCAGGGTCCGAGGTATTCGCACTGGATACGACTTCAGTACCTTAG
	Forward qPCR	GCCGCGCTAGTGGAACTC
	TaqMan Probe	CTGGATACGACTTCAGTAC
	Reverse qPCR	GTGCAGGGTCCGAGGT
siF12	RT	GTCGTATCCAGTGCAGGGTCCGAGGTATTCGCACTGGATACGACCAGAAACTCA
	Forward qPCR	GCCGCGCTAAAGCACTTTAT
	TaqMan Probe	CTGGATACGACCAGAAACT
	Reverse qPCR	GTGCAGGGTCCGAGGT

SUPPLEMENTARY FIGURES

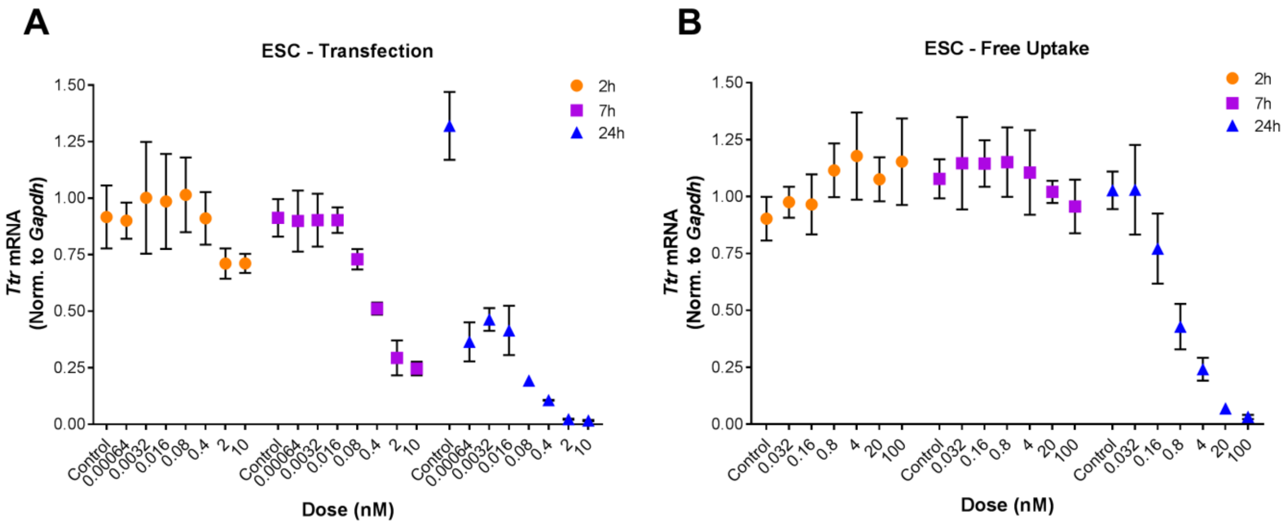

Figure S1: ESC (siTTR-1) GalNAc-siRNA-mediated knockdown of *Ttr* mRNA in primary mouse hepatocytes (PMH) following transfection (**A**) or free uptake (**B**) relative to *Gapdh*. A dose response and time course were carried out for all conditions. Data is represented as mean ± SD, for N = 3.

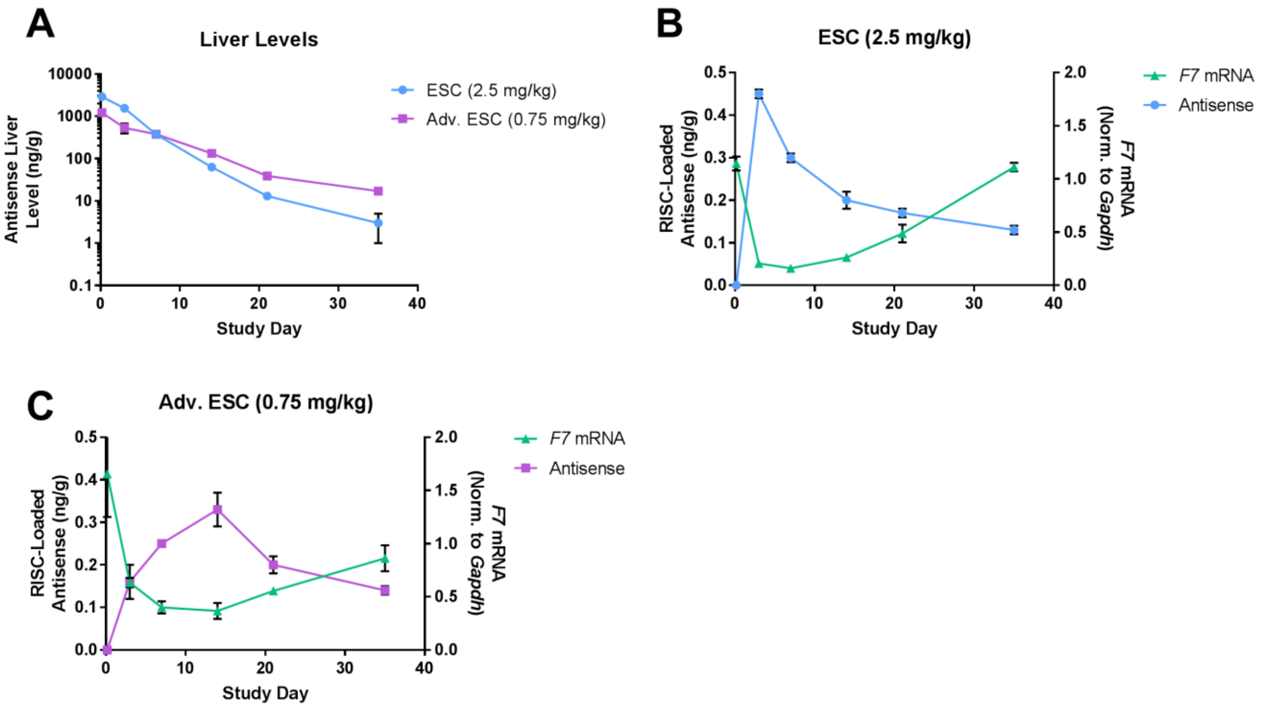

Figure S2: Increased siRNA stability improves knockdown and duration of activity. Cohorts of mice were dosed with ESC (2.5 mg/kg, siF7-1) or Advanced ESC (0.75 mg/kg, siF7-2) siRNA targeting *F7* (**A-C**). Animals were sacrificed after 4 hours and on days 3, 7, 14, 21 and 35 post dose. Antisense siRNA levels (μg/g, μg antisense strand per gram of liver) were measured from total liver (**A**) and plotted on a log10 scale. RISC-loaded antisense siRNA levels were also measured (ng/g, ng of antisense strand per gram of liver) and plotted for ESC (**B**) and Advanced ESC (**C**) siRNAs. *F7* mRNA knockdown was quantified and normalized to *Gapdh* for all samples (**B-C**). Data is represented as mean ± SD, for N = 3.

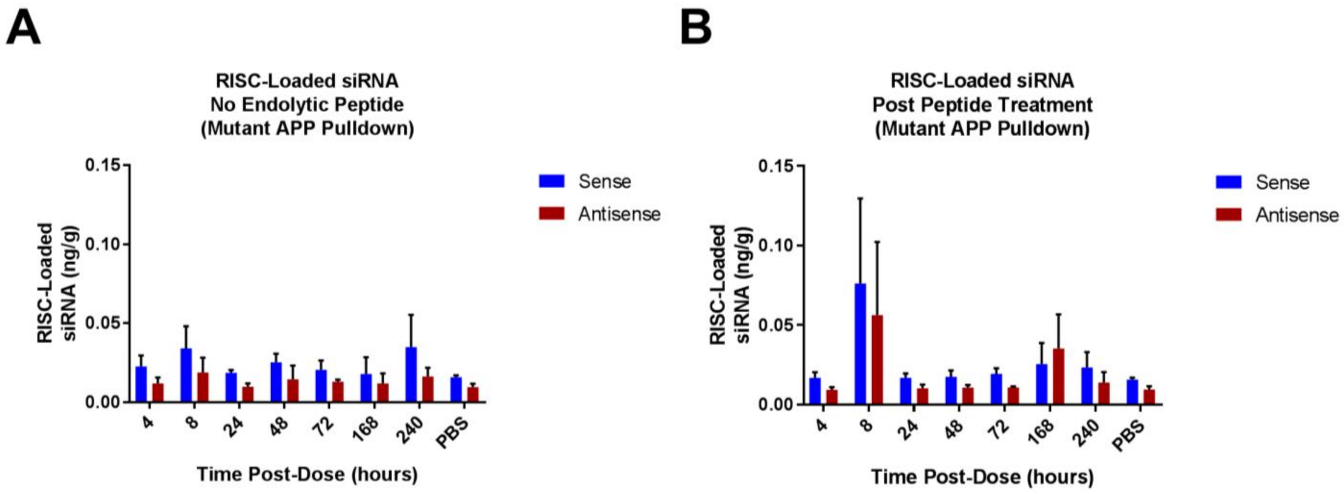

Figure S3: Additional Ago-APP controls for Figure 9. An ESC GalNAc-siRNA (siTTR-1) targeting *Ttr* was dosed SC at 0.5 mg/kg. Half of the mice in the study were treated with 5 mg/kg of the GalNAc-INF7 endolytic peptide dosed SC 15 min after the siRNA dose. Cohorts of mice were sacrificed at several times post-dose (4 hours through day 10). Antisense (red) and sense (blue) strand RISC loading was quantified by RT-qPCR in control (**A**) or endolytic peptide-treated animals (**B**) using a mutant Ago-APP peptide in which all tryptophan residues are mutated to alanine, removing its capacity to bind Argonaute proteins (1). Data is represented as mean ± SD, for N = 3.

SUPPLEMENTARY REFERENCE

1. Hauptmann, J., Schraivogel, D., Bruckmann, A., Manickavel, S., Jakob, L., Eichner, N., Pfaff, J., Urban, M., Sprunck, S., Hafner, M. *et al.* (2015) Biochemical isolation of Argonaute protein complexes by Ago-APP. *Proc Natl Acad Sci U S A*, **112**, 11841-11845.
